# Alkaline-Earth-Catalyzed Dehydrocoupling of Amines and Boranes

**DOI:** 10.1002/anie.201505949

**Published:** 2015-09-11

**Authors:** David J Liptrot, Michael S Hill, Mary F Mahon, Andrew S S Wilson

**Affiliations:** Department of Chemistry, University of Bath Bath BA2 7AY (UK) E-mail: msh27@bath.ac.uk

**Keywords:** amines, alkaline-earth metals, B–N coupling, boron, magnesium

## Abstract

Dehydrocoupling reactions between the boranes HBpin and 9-borabicyclo[3.3.1]nonane and a range of amines and anilines ensue under very mild reaction conditions in the presence of a simple β-diketiminato magnesium *n*-butyl precatalyst. The facility of the reactions is suggested to be a function of the Lewis acidity of the borane substrate, and is dictated by resultant pre-equilibria between, and the relative stability of, magnesium hydride and borohydride intermediates during the course of the catalysis.

Aminoboranes, R_2_N–BR′_2_, find utility in a variety of interesting transformations. Solé and Fernandez, for example, have shown that aminoboranes provide convenient sources of nucleophilic amide anions for reactions with activated alkenes, alkynes, and strained lactones.[1] Extensive work by Suginome et al. has demonstrated that similar reagents enable ready access to iminium cations through their reactions with ketones and aldehydes,[2] while the action of aminodi(boranes), RN(BR_2_)_2_, on ketones has been shown to provide imines through the formation of thermodynamically favored boron–oxygen bonds.[3] However attractive, the more widespread uptake of these applications is hindered by the multistep synthetic routes necessary to yield anything more than a very narrow scope of aminoboranes.

Although some latent dehydrogenative reactivity exists between protic amines and the parent borane, B_2_H_6_, or highly Lewis-acidic dialkylboranes such as 9-borabicyclo[3.3.1]nonane (9-BBN),[4–6] the synthesis of aminoboranes by this route is unreliable and often requires forcing conditions, or fails completely for common borate esters such as pinacol or catecholborane. More reliably, the action of tin–nitrogen[7] and silicon–nitrogen[4, 8] bonds upon boranes and haloboranes yields aminoboranes and the relevant E–X bond. These methods are disfavored, however, by the formation of stoichiometric quantities of the group 14 by-products and, in the case of tin, a toxic waste stream. As a result, most popular synthetic routes to aminoboranes utilize the reaction of lithium amides with BCl_3_.[9] With these limitations in mind, a general and simple dehydrocoupling route to aminoboranes, by the reaction of hydridic B–H and protic N–H bonds, would be highly desirable.

The dehydrocoupling of amine-boranes, R_*n*_NH_3−*n*_⋅BH_3_ (*n*=0, 1, 2), which typically produces oligo- and polyborazane products, has elicited intense recent interest for potential hydrogen storage applications.[10] It is surprising, therefore, that, beyond an isolated example of rhodium-based dehydrocoupling,[11] the sole precedent for the coupling of an amine and a monohydrido borane arises from our earlier report of the reaction between [(HC{(CMe)(N{2,6-*i*Pr_2_C_6_H_3_})}_2_)Ca(NPh_2_)(thf)] and 9-BBN (Scheme 1). In this case the stoichiometric reaction took place through Ca–N/ H–B metathesis to yield the aminoborane and a calcium borohydride.[12] In this contribution we extend this reactivity to a catalytic regime, thus allowing the facile synthesis of aminoboranes from readily available amine and borane precursors.

**Scheme 1:**
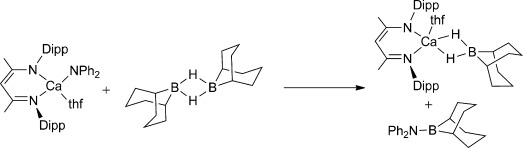
Synthesis of a calcium borohydride (Dipp=2,6-*i*Pr_2_C_6_H_3_).

An initial assessment of the catalytic activity of the β-diketiminato magnesium alkyl and calcium amide species [(HC{(CMe)(N{2,6-*i*Pr_2_C_6_H_3_})}_2_)MgBu] (**I**) and [(HC{(CMe)(N{2,6-*i*Pr_2_C_6_H_3_})}_2_)Ca{N(SiMe_3_)_2_}(thf)] (**II**) was undertaken for the reaction of diethylamine and pinacolborane (HBpin). Although a 10 mol % loading of both precatalysts proved to be competent for this dehydrocoupling at ambient temperature, the magnesium-mediated process provided superior reactivity and an effective stoichiometric conversion into *N*,*N*-diethyl-4,4,5,5-tetramethyl-1,3,2-dioxaborolan-2-amine. Encouraged by this observation, we undertook a study into the scope of the magnesium-catalyzed dehydrocoupling reactivity. Table 1 summarizes an investigation into the ability of **I** to catalyze the dehydrocoupling of a range of primary and secondary amines and anilines with both HBpin and 9-BBN. The weak Lewis acid HBpin was observed to couple readily with aromatic and aliphatic amines of varying bulkiness (entries 1–8), but failed to react with the very bulky HN(SiMe_3_)_2_ (entry 9). Most reactions occurred with a pronounced bubbling and reached completion at room temperature in less than a day. Reactions of the more Lewis-acidic 9-BBN were more dependent on the identity of the amine. While less sterically congested alkyl amines coupled readily (entry 10), reactions with more bulky substrates (entry 11) were slower and comparable to the background reactions. Similarly, smaller anilines coupled at rates significantly in excess of the background reactions (entry 12), while bulkier anilines reverted to the uncatalyzed rate (entries 13 and 14).

**Table 1:** Study of the scope of boron–nitrogen dehydrocoupling catalyzed by I

Entry	Borane	Amine	Product	*t* [h]	Conv. [%]^[c]^
**1**	HBpin	*n*BuNH_2_	pinBNH*n*Bu	<1^[a]^	99
**2**		*t*BuNH_2_	pinBNH*t*Bu	<1^[a]^	99
**3**		PhNH_2_	pinBNHPh	<1^[a]^	99
**4**		DippNH_2_	pinBNHDipp	<1^[a]^	99
**5**		PhN(H)Me	pinBN(Me)Ph	<1^[a]^	99
**6**		(CH_2_)_4_NH	pinBN(CH_2_)_4_	<1^[a]^	99
**7**		Et_2_NH	pinBNEt_2_	<1^[a]^	99
**8**		Ph_2_NH	pinBNPh_2_	<1^[a]^	99
**9**		(Me_3_Si)_2_NH	No reaction	144^[b]^	0
**10**	9-BBN	*n*BuNH_2_	R_2_BNH*n*Bu	<1^[a]^	99
**11**		*t*BuNH_2_	R_2_BNH*t*Bu	144^[b]^	83
**12**		PhNH_2_	R_2_BNHPh	12^[a]^	99
**13**		DippNH_2_	R_2_BNHDipp	144^[b]^	80
**14**		Ph_2_NH	R_2_BNPh_2_	144^[b]^	73

Reaction conditions: **I** (0.01 mmol) with amine (0.1 mmol) and borane (0.1 mmol) in C_6_D_6_ (0.5 mL). [a] At room temperature. [b] At 60 °C. [c] Determined by ^1^H NMR spectroscopy.

Aminodi(boranes) were readily accessible by adjustment of the reaction stoichiometry between primary amines and both HBpin and 9-BBN. Reactions performed between two molar equivalents of both borane substrates and one equivalent of *n*-butylamine provided quantitative conversions into the bis(boryl)ated amines (Table 2, entries 1 and 4), albeit with gentle heating and slightly extended reaction times compared to those for aminoborane formation. Although *tert*-butylamine also coupled twice with pinacolborane (entry 2) neither borane showed any sign of twofold dehydrocoupling with aniline (entries 3 and 5).

**Table 2:** Catalytic boron–nitrogen dehydrocoupling catalyzed by I

Entry	Amine	Ratio	Product	*t* [days]	Conv. [%]^[c]^
HBpin					
1	*n*BuNH_2_	2:1	(PinB)_2_N*n*Bu	1^[b]^	99
2	*t*BuNH_2_	2:1	(PinB)_2_N*t*Bu	<1^[a]^	99
3	PhNH_2_	2:1	PinBNHPh	2^[b]^	99
9-BBN					
4	*n*BuNH_2_	2:1	R_2_BHN*n*Bu	1^[a]^	99
5	PhNH_2_	2:1	R_2_BNHPh	2^[b]^	99

Reaction conditions: **I** (0.01 mmol) with amine (0.1 mmol) and borane (0.2 mmol) in C_6_D_6_ (0.5 mL). [a] At room temperature. [b] At 60 °C. [c] Determined by ^1^H NMR spectroscopy.

The nature of these variations was investigated through a series of stoichiometric reactions. An NMR-scale reaction performed between 2,6-di-isopropylphenyl aniline (DippNH_2_) and **I**, with subsequent addition of HBpin, yielded a ^11^B NMR spectrum (*δ*=32.1 ppm) which was indicative of a single three-coordinate boron environment. The corresponding ^1^H NMR spectrum was less informative and comprised a complex set of signals in the regions attributable to the pinacol methyl and isopropyl resonances. The origin of these observations was resolved through the isolation of single crystals of **1**, at −38 °C from toluene, suitable for X-ray diffraction analysis (Figure 1 a). The compound **1** is a magnesium anilido(pinacol)borane and is the first structurally characterized species of this class. Whilst a variety of magnesium amidoborane complexes have been structurally characterized, such derivatives contain four-coordinate boron centers and B–H⋅⋅⋅Mg agostic-type interactions.[13] Notably, a donor interaction with a pinacolate oxygen atom raises the Mg coordination number of **1** to four. The complexity of the pinacol methyl region in the ^1^H NMR spectrum can thus be rationalized as a result of the persistence of the Mg–O bond in the nondonor deuterobenzene solvent.

**Figure 1:**
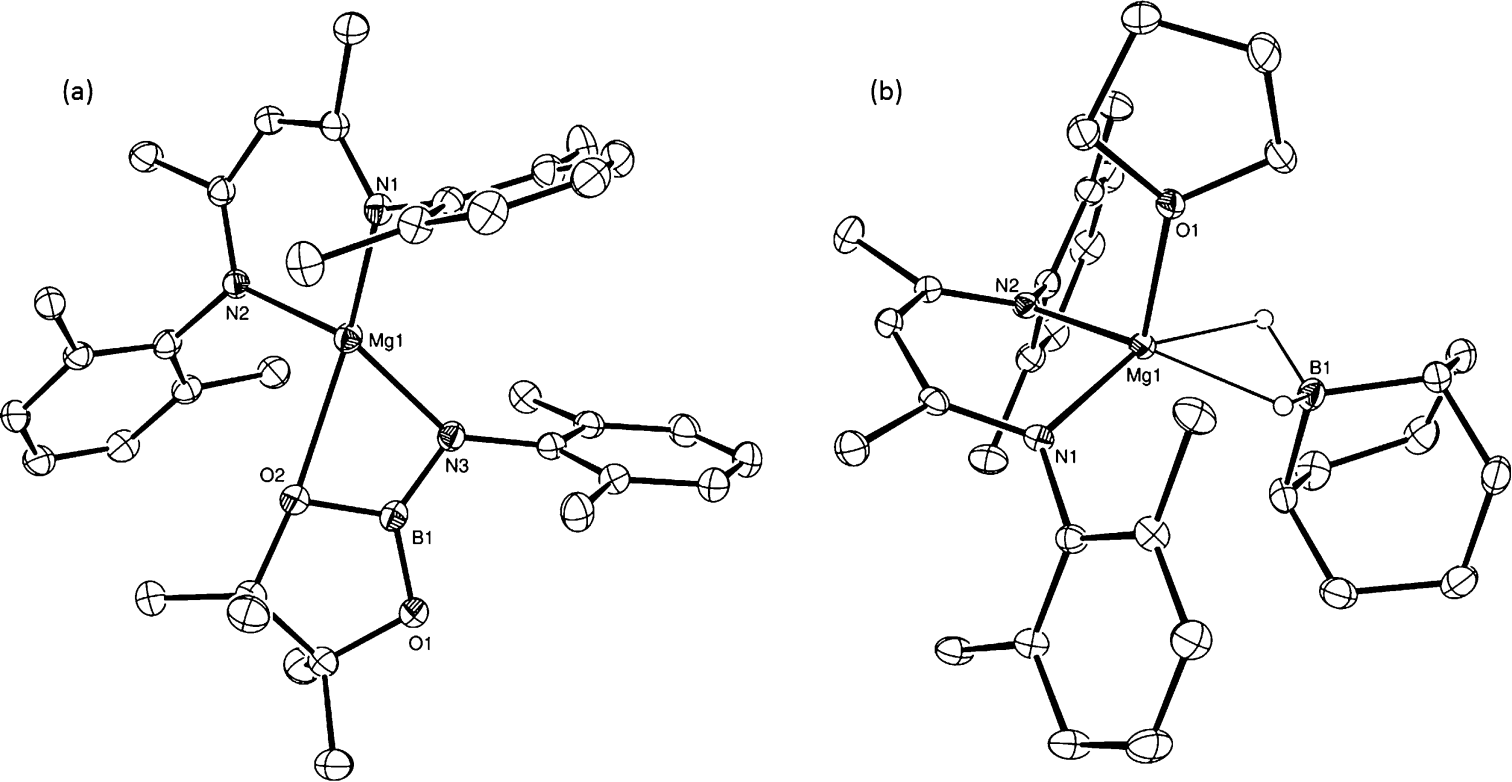
ORTEP representations of the compounds 1 (a) and 2 (b; 30 % probability ellipsoids). Isopropyl methyl groups and hydrogen atoms omitted for clarity. Selected bond lengths [Å] and angles [°]: (1) Mg1–O2 2.3737(18), Mg1–N3 2.003(2), O2–B1 1.418(3), B1–N3 1.379(4), N3–C30 1.423(3); N1-Mg1-N2 94.72(8), Mg1-O2-B1 80.94(14), O2-B1-N3 116.8(2), B1-N3 Mg1 96.96(15), B1-N3-C30 120.6(2); (2) Mg1–B1 2.3092(15), Mg1–O1 2.0556(9), Mg1–H1a 1.932(14), Mg1–H1b 1.929(15), B1–C35 1.618(2), B1–C39 1.6026(19); N1-Mg1-N2 93.48(4), N1-Mg1-B1 125.62(5), C35-B1-C39 107.44(11).

A similar reaction performed with half an equivalent of the 9-BBN dimer yielded a ^11^B NMR spectrum comprising a single resonance at *δ*=27.5 ppm, which is attributable to DippN(H)BR_2_. The corresponding ^1^H NMR spectrum indicated the clean formation of two species, identified as DippN(H)BR_2_ and the previously reported dimeric magnesium hydride [(HC{(CMe)(N{2,6-*i*Pr_2_C_6_H_3_})}_2_)MgH]_2_.[14] Notably, a similar reaction performed with a stoichiometric quantity of 9-BBN provided two new boron-containing compounds. While DippN(H)BR_2_ was again observed to form with complete consumption of the aniline, an additional species (**2**) was characterized by a broadened triplet resonance at *δ*=−13.8 ppm in the ^11^B NMR spectrum. A further X-ray diffraction analysis confirmed the identity of **2** (Figure 1 b) as a pseudo-square-pyramidal magnesium borohydride reminiscent of the calcium-centered product illustrated in Scheme 1. Compound **2** is thus closely related to the more expansive class of magnesium borohydrides containing [BH_4_]^−^ anions, including Mountford’s β-diketiminato- magnesium tetra(hydrido)borate, [(HC{(CMe)-(N{2,6-*i*Pr_2_C_6_H_3_})}_2_)Mg(BH_4_)].[15] More pertinently, these observations suggest that, when in excess, coordination of the Lewis-acidic 9-BBN to the magnesium hydride and formation of **2** is competitive with the deprotonation of DippN(H)BR_2_.

The validity of these deductions was investigated through a kinetic study of the reactions of *N*-methylaniline with HBpin and 9-BBN catalyzed by **I**. In line with the data collated in Table 1, the turnover frequency (TOF) of the HBpin-based reaction [9935(1353) h^−1^] was found to be significantly higher than for the reaction with 9-BBN [26.4(28) h^−1^]. While a background reaction perfomed between HBpin and *N*-methylaniline provided no evidence of dihydrogen evolution, this latter TOF value also surpassed that of an uncatalyzed reaction, utilizing 9-BBN, by an order of magnitude.

The dehydrocoupling of HBpin with *N*-methylaniline conformed to global first-order kinetics (see Figure S1 in the Supporting Information), which varied with a second-order dependence on catalyst concentration (see Figure S2 in the Supporting Information). Pseudo-first-order experiments were indicative of zero- and first-order rate dependences on [PhNH(Me)] and [HBpin], respectively and the rate law shown as Equation (1).


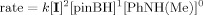
(1)

Dehydrocoupling reactions performed with 9-BBN also provided linear first-order rate plots for more than three half-lives at a range of loadings of **I** (see Figure S4 in the Supporting Information). In this case, however, a plot of the resultant observed rate constants was found to vary linearly with the square root of [**I**], thus indicating a one-half-order dependence on the precatalyst concentration (see Figure S5 in the Supporting Information). The partial reaction order with respect to the borane substrate was deduced through a pseudo-first-order methodology in which the consumption of the limiting reagent with time was found to be indicative of an inverse dependence on [9-BBN]. Consistent with this apparent substrate inhibition, a similar approach utilizing an excess of 9-BBN suppressed the reaction to such an extent that there was no observable consumption of the aniline substrate. We interpret this inhibition to indicate the persistence of a borohydride species similar to **2** under reaction conditions comprising a large excess of the Lewis-acidic borane. These experimental limitations notwithstanding, the global first-order kinetics of the reaction infer a second-order dependence on [PhNH(Me)] and the formulation of a rate law for this reaction of the form shown in Equation (2).


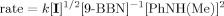
(2)

The activation parameters for both reactions were deduced through variable-temperature Arrhenius and Eyring analyses (Table 3). A now considerable literature precedent has highlighted the impact of entropic effects on the rate-determining energetics during many reactions catalyzed by group 2 reagents.[16] Whilst HBpin provided a negative entropy of activation indicative of a highly ordered rate-determining transition state, the reaction of 9-BBN and *N*-methylaniline was observed to entail a significantly positive value [20.0(25) cal mol^−1^ K^−1^].

**Table 3:** Activation parameters for the catalytic dehydrocoupling of *N*-methylaniline with HBpin and 9-BBN mediated by I

	*E*_a_ [kcal mol^−1^]	Δ*H*^≠^ [kcal mol^−1^]	Δ*S*^≠^ [cal mol^−1^ K^−1^]	Δ*G*^≠^_298_ [kcal mol^−1^]
HBpin	9.8(7)	9.2(7)	−47.9(25)	23.5
9-BBN	29.5(7)	29.9(7)	20.0(25)	23.0

These data indicate a considerable mechanistic variance across the two reactions. We infer that the kinetic profile of the reaction involving 9-BBN reflects a pre-equilibrium between a magnesium borohydride, similar to **2**, and a dimeric magnesium hydride (indicated as **A** and **B**, respectively, in Scheme 2). The stability of the former species as an off-loop resting state of the reaction effectively limits the magnesium hydride available to engage in onward reactivity. We suggest that the notable half-order dependence on precatalyst concentration, [**I**], the inhibition of the reaction rate by increasing borane concentration and the implied second-order behavior with respect to [PhNH(Me)] implicates a monomer–dimer equilibrium between the dinuclear species **B** and an adduct of the weakly basic aniline (**C**) in the rate-limiting process of the reaction. The onward trajectory of the catalysis is then predicated on hydrogen elimination from **C** and anilide formation (**D**). We suggest that subsequent and facile B–N coupling occurs through nucleophilic attack of the magnesium-bound anilide on the Lewis acidic 9-BBN substrate and rapid β-hydride transfer to the magnesium center via the borate intermediate **E** in a sequence reminiscent of that deduced by both Sadow and co-workers and Sarazin and co-workers for a closely related silane–amine dehydrocoupling and our previous observations of group 2-centered amine–borane dehydrocoupling catalysis.[17, 13]

**Scheme 2:**
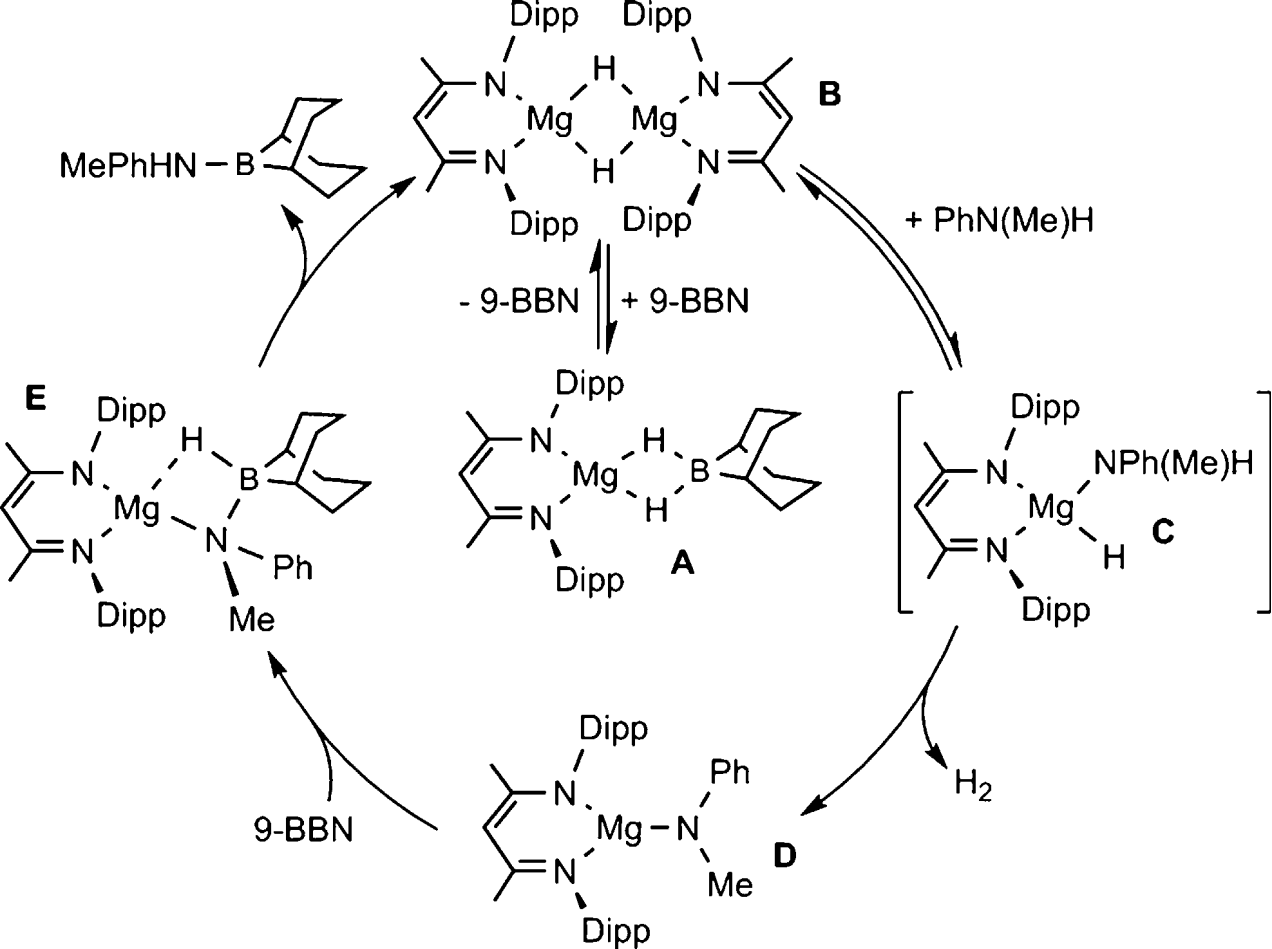
Proposed mechanism for Mg-catalyzed dehydrocoupling of amines and boranes.

For the reaction derived from HBpin, we suggest that the formation of an off-cycle borohydride similar to **A** is disfavored by the significantly reduced Lewis acidity of the borate ester substrate. Although facile aniline protonolysis will ensue under this regime, direct nucleophilic attack by the anilide on HBpin is also likely to be disfavored by the attenuated Lewis acidity of the borane. We suggest, therefore, that the second-order dependence on [**I**] implicates the action of a further borohydrido magnesium species akin to **A**, and is necessary to deliver the HBpin substrate to the magnesium anilide. In this case, it is feasible that the formation of the [H_2_Bpin]^−^ anion will facilitate the assembly of a bimetallic intermediate through the augmentation of the Lewis basicity of the pinacolate oxygen centers and a consequently enhanced facility for intermolecular Mg-O coordination.

In summary, we have shown that high conversions to amineboranes may be achieved through the magnesium- and calcium-catalyzed deydrocoupling of readily available amines and boranes. Albeit currently restricted to cyclic borane substrates, the facility of the catalysis is shown to be profoundly affected by electronic variations within the borane coupling partner.

## Experimental Section

CCDC 1408761 and 1408762 contain the supplementary crystallographic data for this paper. These data can be obtained free of charge from The Cambridge Crystallographic Data Centre.

## Supporting Information

As a service to our authors and readers, this journal provides supporting information supplied by the authors. Such materials are peer reviewed and may be re-organized for online delivery, but are not copy-edited or typeset. Technical support issues arising from supporting information (other than missing files) should be addressed to the authors.

miscellaneous_information
