# Supplementary material for: Alkaline-Earth-Catalyzed Dehydrocoupling of Amines and Boranes
Source: Angew Chem Int Ed Engl. 2015 Sep 11;54(45):13362–5. doi: 10.1002/anie.201505949 (PMC4674969; doi:10.1002/anie.201505949)
Supplement: Supplementary file 1 — miscellaneous_information [file anie0054-13362-sd1.pdf]

Angewandte Chemie
Eine Zeitschrift der Gesellschaft Deutscher Chemiker

Supporting Information

Alkaline-Earth-Catalyzed Dehydrocoupling of Amines and Boranes

David J. Liptrot, Michael S. Hill, Mary F. Mahon, and Andrew S. S. Wilson*

anie_201505949_sm_miscellaneous_information.pdf

General experimental procedures

All reactions dealing with air- and moisture-sensitive compounds were carried out under an argon atmosphere using standard Schlenk line and glovebox techniques in an MBraun Labmaster glovebox at O_2, H_2O < 0.1 ppm. NMR experiments using air-sensitive compounds were conducted in J. Youngs tap NMR tubes prepared and sealed in a glovebox under argon. All NMR data were acquired on a Bruker 300 Ultrashield™ for ^{1}H (300 MHz), ^{13}C{^{1}H} (75.48 MHz) and ^{11}B (96.3 MHz) spectra at room temperature or a Bruker 400 Ultrashield™ for ^{1}H (400 MHz) and ^{13}C{^{1}H} (125.76 MHz) spectra. ^{1}H/^{13}C NMR spectra were referenced using residual solvent resonances. Elemental analyses of all moisture- and air-sensitive compounds were performed by Stephen Boyer of London Metropolitan Enterprises. Solvents for air- and moisture-sensitive reactions were provided by an Innovative Technology Solvent Purification System. C_6D_6 and toluene-d_8 were purchased from Fluorochem and dried over molten potassium prior to vacuum transfer into a sealed ampoule and storage in the glovebox under argon. ArNC(Me)CHC(Me)NHAr (Ar =2,6-di-iso-propylphenyl), [HC{(Me)CN(2,6-^{i}Pr$_2$C$_6$H$_3$)}$_2$Mg^{n}Bu] (**I**) and [HC{(Me)CN(2,6-^{i}Pr$_2$C$_6$H$_3$)}$_2$Ca{N(SiMe$_3$)$_2$}(THF)] (**II**) were synthesized by literature procedures.[1-3]

[CH{C(Me)N(Dipp)}$_2$}Mg{N(Dipp)Bpin}], 1

To a toluene solution (3 mL) of **I** (50 mg, 0.1 mmol) was added DippNH$_2$ (18.85 µL, 0.1 mmol) and the resultant solution, which initially bubbled, was allowed to sit for 1 h. After this time pinacol(borane) (14.4 µL, 0.1 mmol) was added and a bubbling noted again. After 1 h., the solvent was removed *in vacuo* to incipient crystallisation and chilled to –34°C yielding material suitable for crystallographic characterisation (27 mg, 34%, m.p. 69 °C (dec)). ^{1}H NMR (300MHz, Tol-d_8) δ ppm 0.81 (s, 6 H, OC(C<u>H</u>$_3$)$_2$) 0.90 (d, J = 6.78 Hz, 6 H, CH(C<u>H</u>$_3$)$_2$) 0.97 (s, 6 H, OC(C<u>H</u>$_3$)$_2$) 1.02 – 1.08 (m, 12 H, CH(C<u>H</u>$_3$)$_2$) 1.17 (d, J = 6.40 Hz, 6 H, CH(C<u>H</u>$_3$)$_2$) 1.18 (d, J = 6.22 Hz, 6 H, CH(C<u>H</u>$_3$)$_2$) 1.35 (d, J = 6.78 Hz, 6 H, CH(C<u>H</u>$_3$)$_2$) 1.61 (s, 6 H, CH(CC<u>H</u>$_3$)$_2$) 3.12 – 3.39 (m, 6 H, C<u>H</u>(CH$_3$)$_2$) 4.86 (s, 1 H, C<u>H</u>(CCH$_3$)$_2$) 7.02 - 7.10 (m, 9 H, Ar<u>H</u>); ^{13}C NMR (75 MHz, Tol-d_8) δ ppm 171.0, 146.4, 143.6, 142.3, 141.2, 126.1, 124.1, 123.3, 120.7, 96.4, 83.5, 83.1, 29.3, 28.9, 28.7, 25.4, 25.3, 25.2, 24.9, 24.6; ^{11}B NMR (96 MHz, Tol-d_8) δ ppm 26.8 (s). Infrared (KBr disc, υ cm^{-1}) 3062, 2962, 2869, 1661, 1622, 1551, 1511, 1462, 1439, 1382, 1364, 1326, 1263, 1218, 1163; Anal. Calcd. for $C_{47}H_{70}BMgN_3O_2$: C, 75.85; H, 9.48 N, 5.65%. Found: C, 75.65; H, 9.56; N, 5.77%.

[CH{C(Me)N(Dipp)}$_2$}Mg(THF)(H$_2$B{CHCH$_2$CH$_2$CH$_2$}$_2$)], 2

To a toluene solution (3 mL) of **I** (50 mg, 0.1 mmol) was added DippNH$_2$ (18.85 µL, 0.1 mmol) and the resultant solution, which initially bubbled, was allowed to sit for 1 h. After this time 9-BBN (12.2 mg, 0.1 mmol) was added and a bubbling noted again. After 1 h., the solvent was removed *in vacuo* to incipient crystallisation and was chilled to –34°C yielding a powder. Three drops of THF were thus added and chilling to –34 °C yielded material suitable for crystallographic characterisation (52 mg,

82%, m.p. 130 °C (dec)). ^{1}H NMR (300MHz, d_8-THF) δ ppm 0.47 (s, 2H, R_2B-$\underline{H}_2$) 1.04 – 1.31 (m, 26 H, $CH(C\underline{H}_3)_2$ and 9-BBN) 1.21 (d, J = 6.78 Hz, 12 H, $CH(C\underline{H}_3)_2$) 1.66 (s, 6 H, $CH(CC\underline{H}_3)_2$) 3.11 (m, 4 H, $C\underline{H}(CH_3)_2$) 4.90 (s, 1 H, $C\underline{H}(CCH_3)_2$) 7.02 - 7.10 (m, 6 H, Ar$\underline{H}$); ^{13}C NMR (75 MHz, d_8-THF) δ ppm 170.0, 145.9, 143.1, 125.9, 124.4, 95.5, 68.4, 35.1, 28.9, 26.6, 26.3, 25.3, 25.1, 24.7, 24.1; ^{11}B NMR (96 MHz, d_8-THF) δ ppm –17.4 (t, $^{1}J_{BH}$ = 60 Hz, $R_2\underline{B}$-H_2-Mg). Infrared (KBr disc, υ cm^{-1}) 3060, 2961, 2923, 2866, 2826, 2043, 1662, 1542, 1512, 1463, 1436, 1400, 1364, 1313, 1261, 1175, 1106, 1019. Anal. Calcd. for $C_{37}H_{57}BMgN_2$: C, 78.76; H, 10.17; N, 4.96%. Found: C, 79.11; H, 9.80; N, 4.89%. Analysis was performed on a sample of the isolated single crystals which had been subjected to high vacuum at 60 °C resulting in the removal of both the occluded toluene and the coordinated molecule of THF.

NMR and IR Spectra: Compounds 1 and 2

Figure S1: Compound **1**, ^{1}H NMR

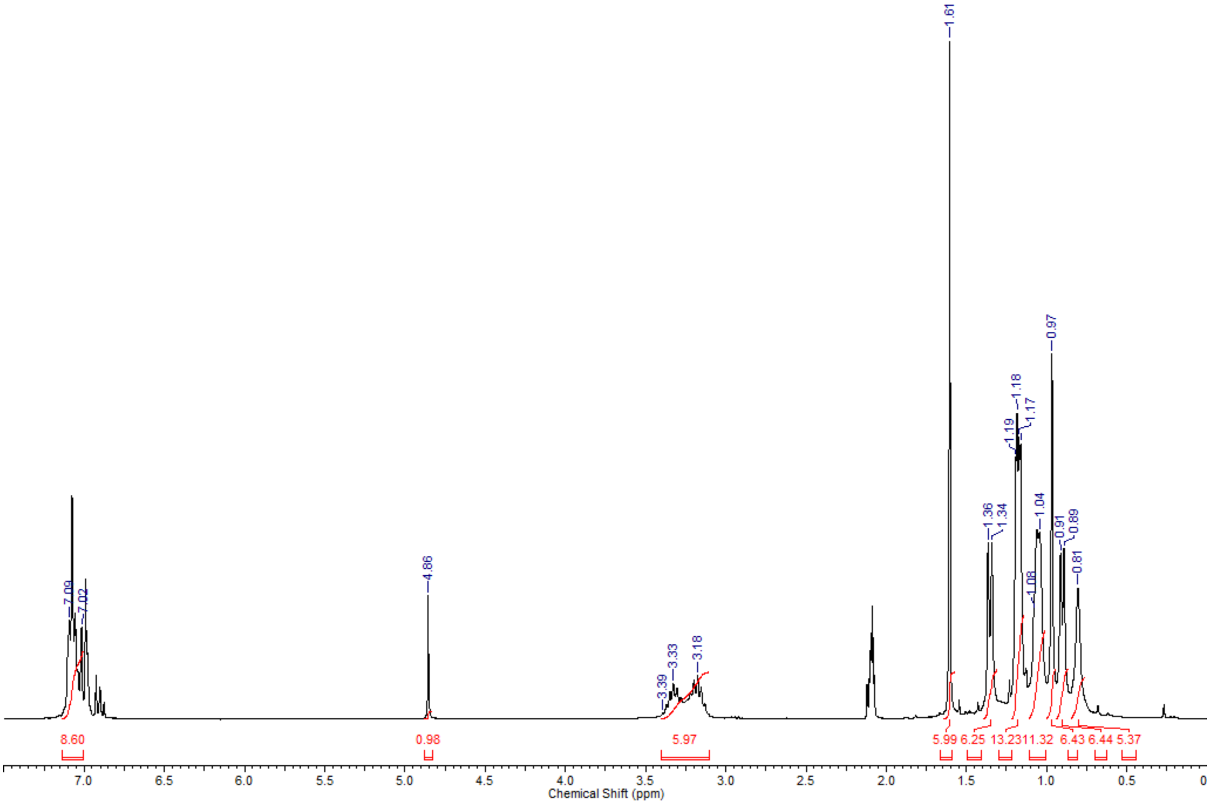

Figure S2: ^{13}C NMR (**1**)

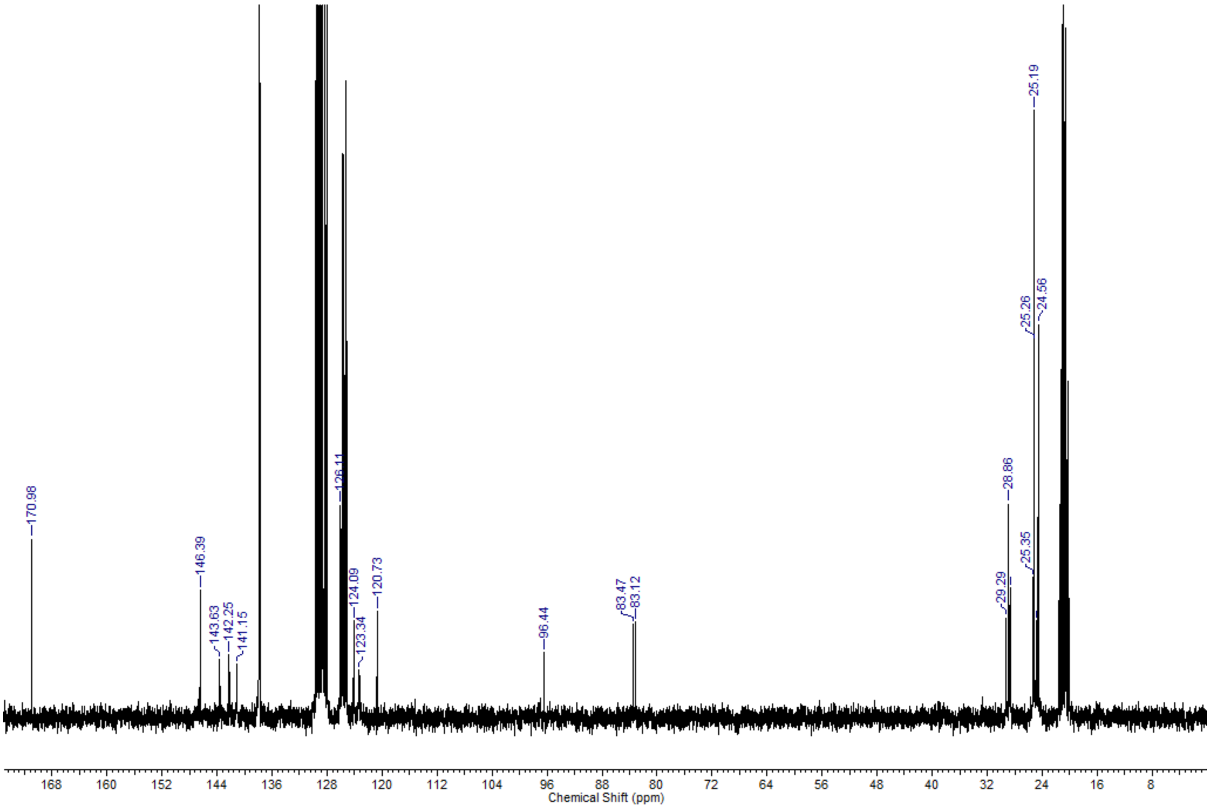

Figure S3: ^{11}B NMR (**1**)

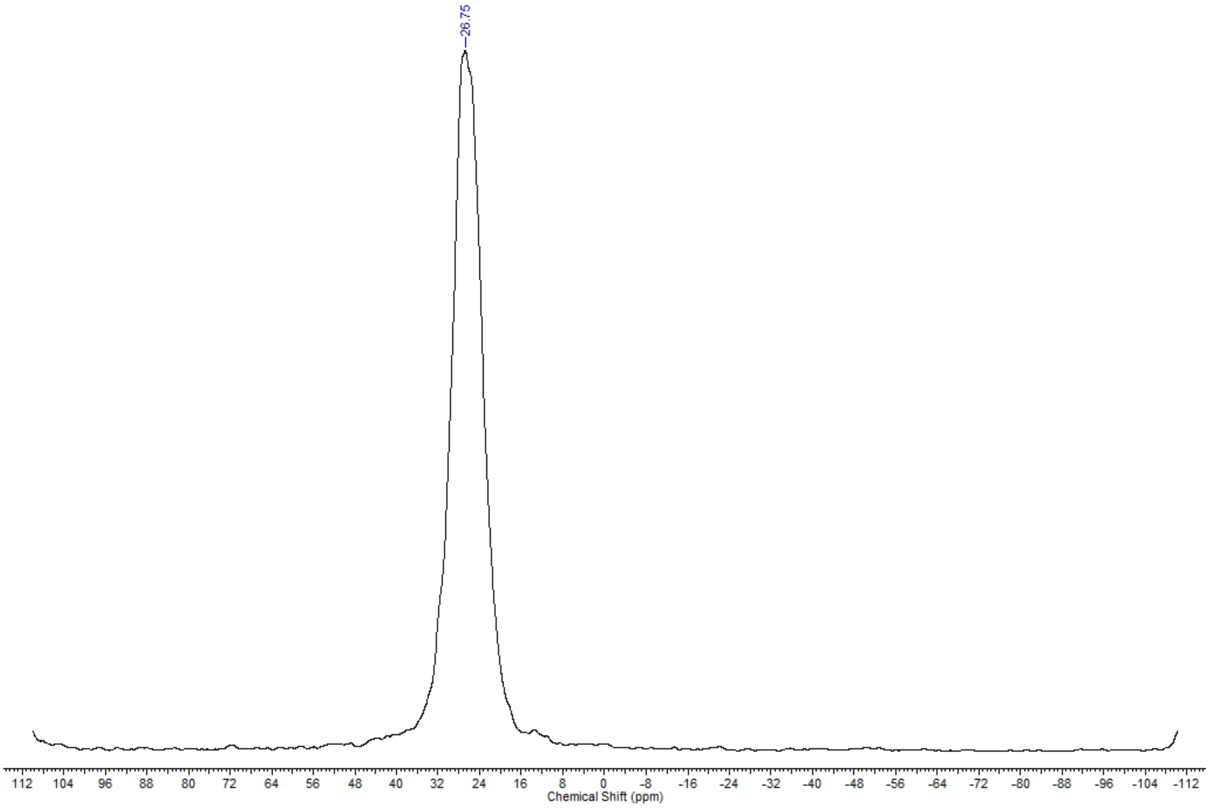

Figure S4: Infra-red (**1**)

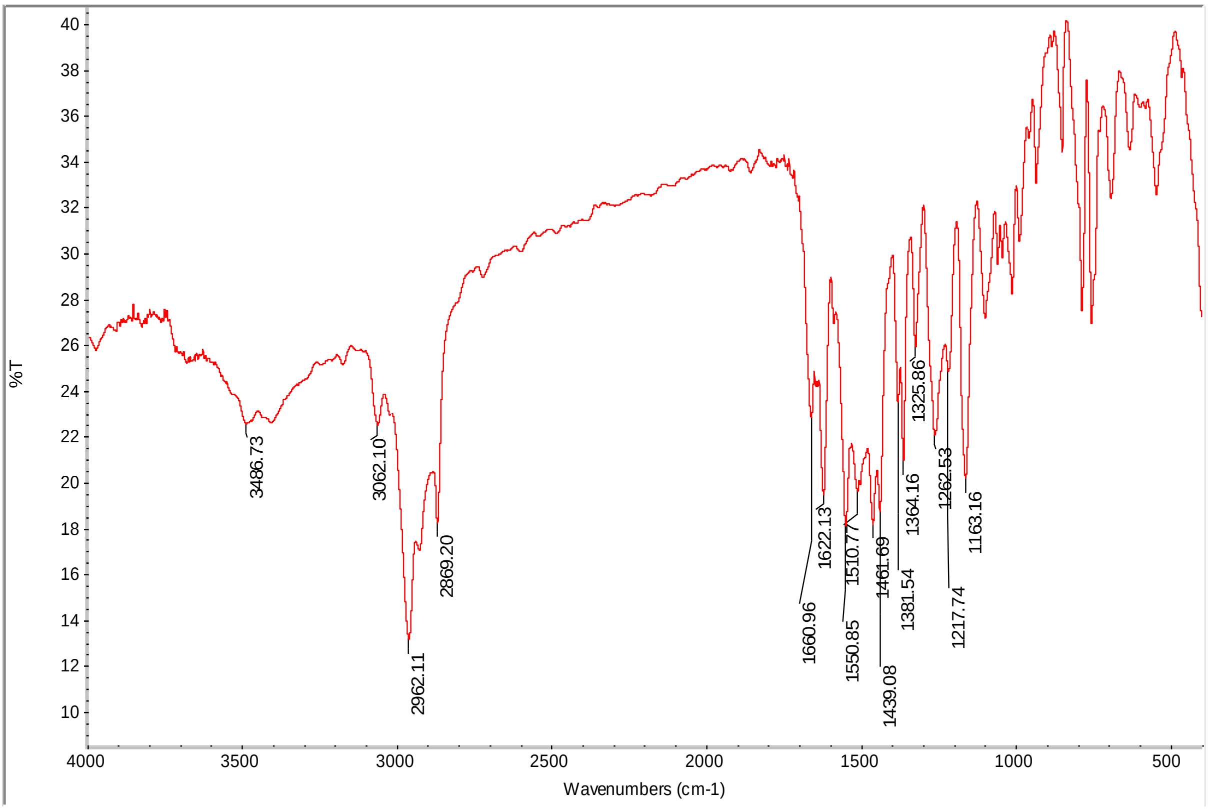

Figure S5: Compound **2**, ^{1}H NMR

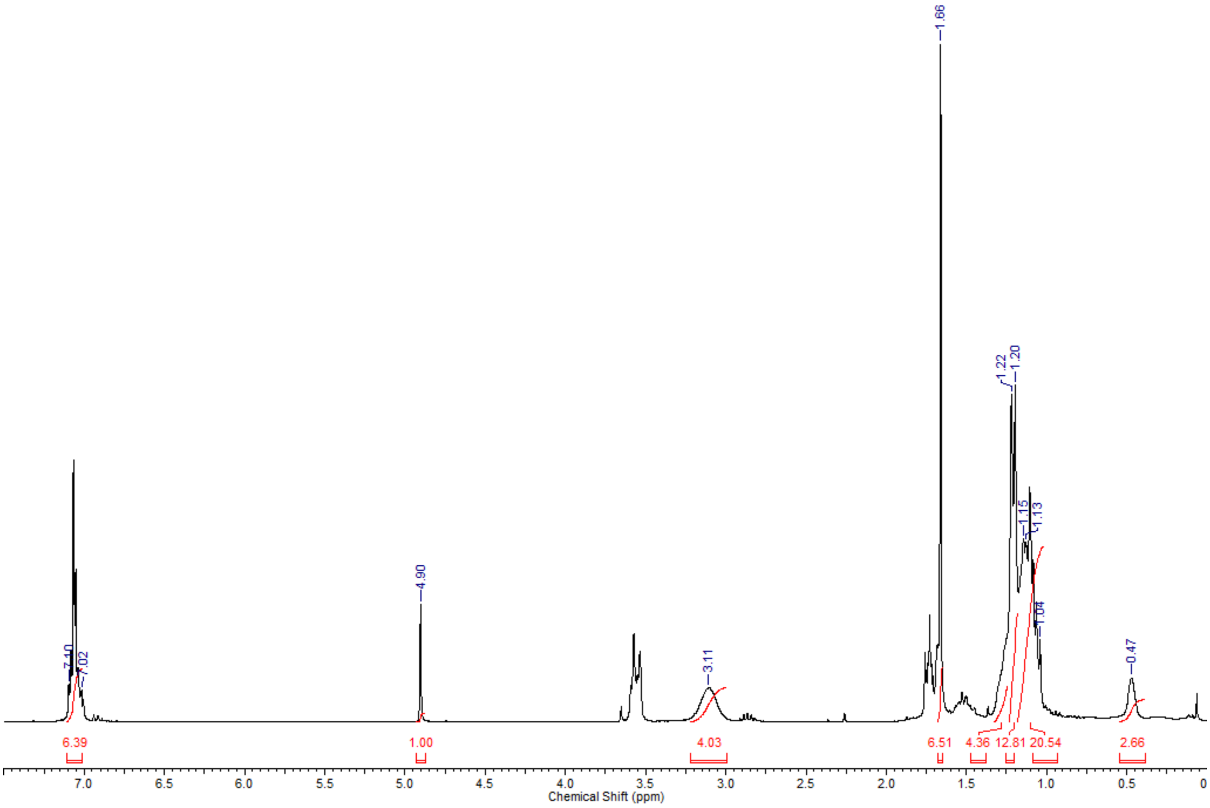

Figure S6: ^{13}C NMR (**2**)

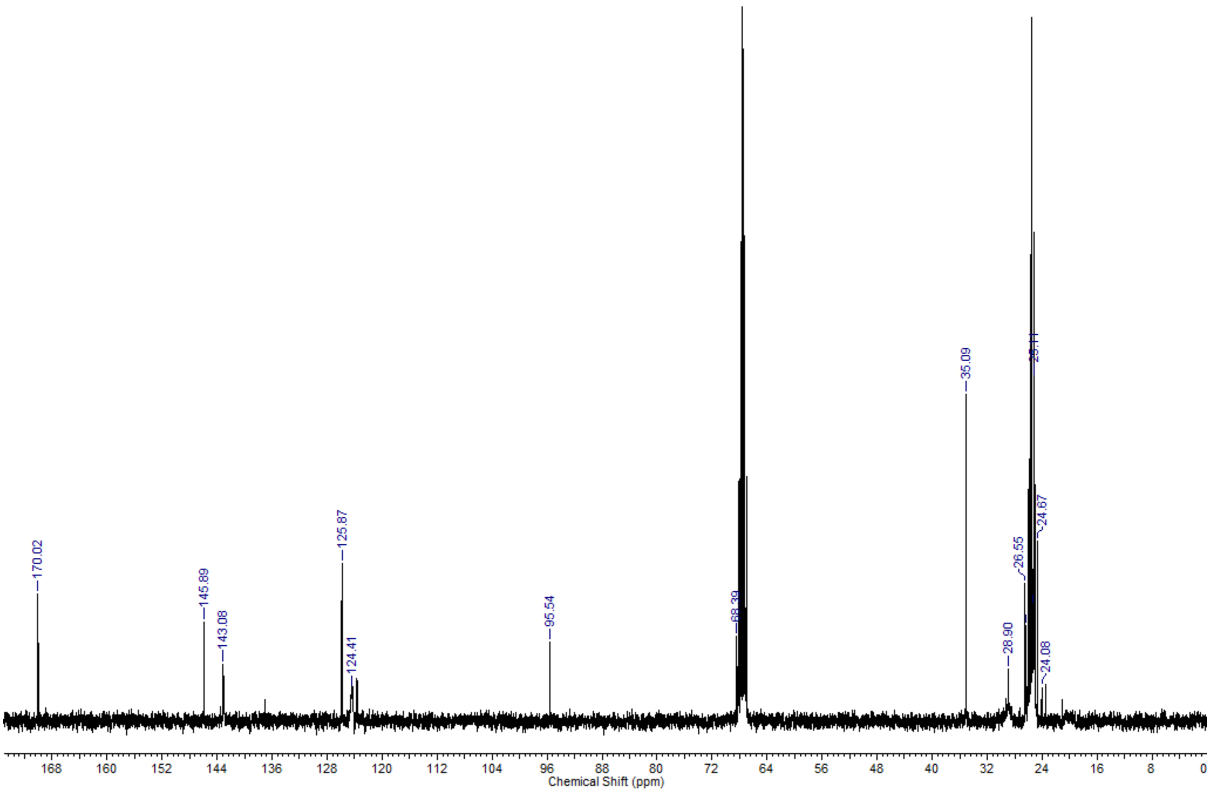

Figure S7: ^{11}B NMR (**2**)

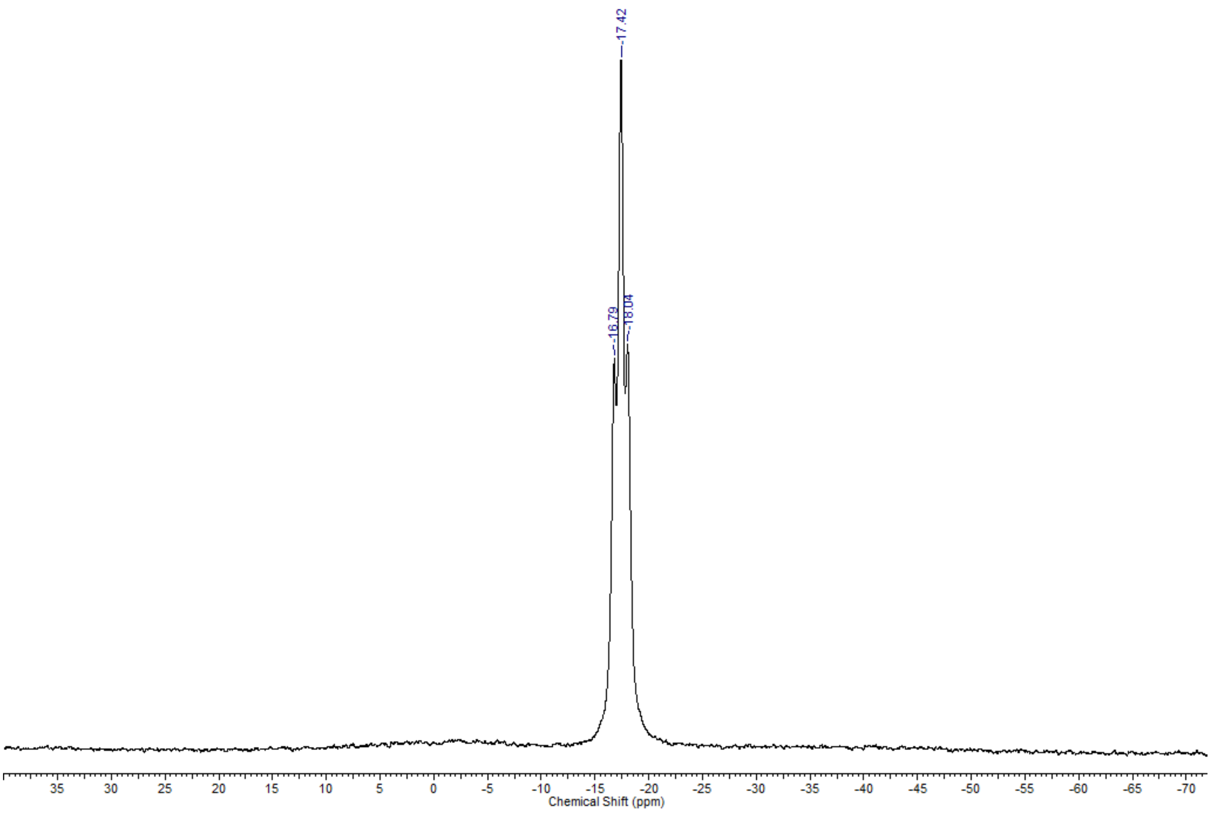

Figure S8: Infra-red (**2**)

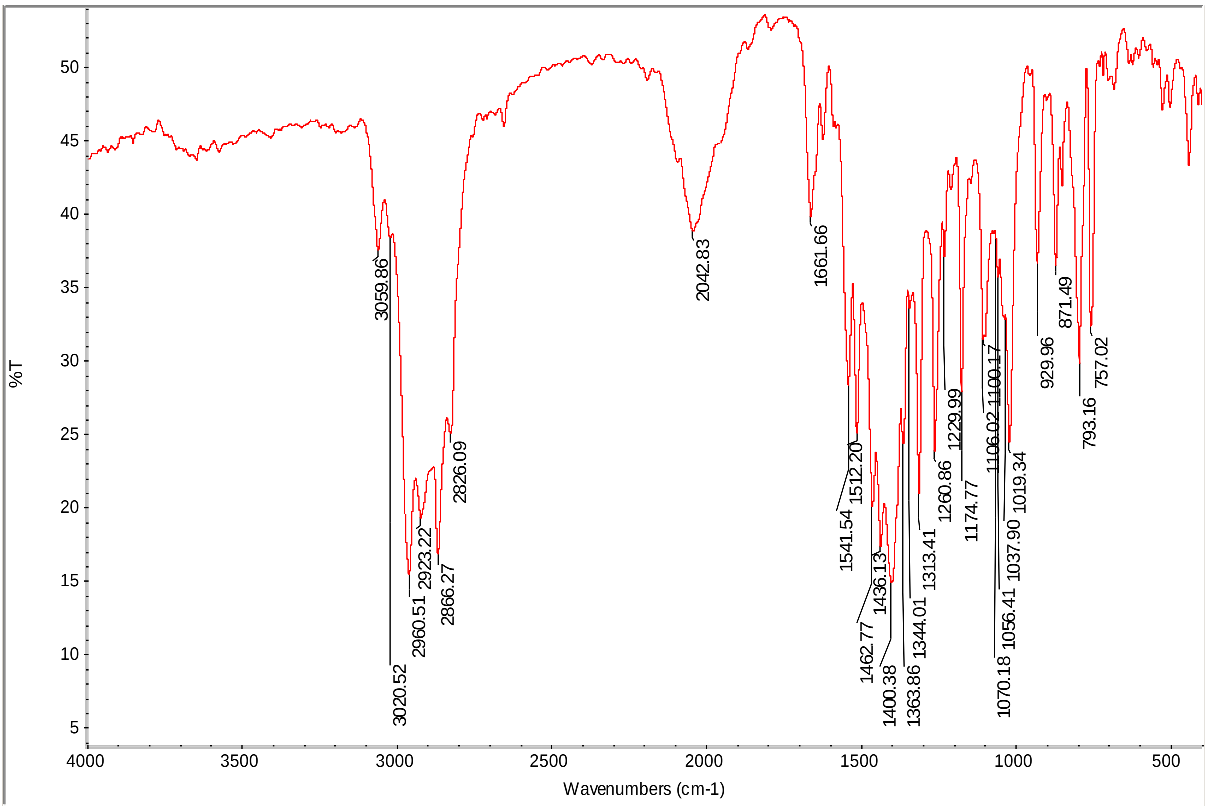

Catalytic NMR experiments

The relevant amounts of dry substrates, catalyst and deuterated solevent were directly added to a Youngs tap NMR tube under inert atmosphere. All liquids were measured with Eppendorf pipettes. A homogenous solution was obtained by repeatedly turning the tube upside down. Aminoboranes were synthesized according to conditions noted in Tables 1 and 2 and were identified by comparison to literature data. Novel compounds are characterized hereafter.

***n*-BuN(H)Bpin** ^{1}H NMR (300 MHz, benzene-*d*$_6$) δ ppm 2.92 (q, *J*=6.8 Hz, 2 H), 2.07 (br. s, 1 H), 1.19 - 1.23 (m, 4 H), 1.10 - 1.17 (m, 12 H), 0.81 ppm (t, *J*=7.5 Hz, 3 H); ^{13}C NMR (benzene-d$_6$,75MHz): δ ppm 82.1, 41.4, 36.4, 25.2, 20.3, 14.4; ^{11}B NMR (96 MHz, benzene-*d*$_6$) δ ppm 28.0 (s).

***t*-BuN(H)Bpin** ^{1}H NMR (300 MHz, benzene-*d*$_6$) δ ppm 1.12 (s, 12 H) 1.21 (s, 9 H); ^{13}C NMR (benzene-d$_6$,75MHz): δ ppm 81.7, 32.6, 25.3, 25.1; ^{11}B NMR (96 MHz, benzene-*d*$_6$) δ ppm 27.33 (s).

***t*-BuN(Bpin)$_2$** ^{1}H NMR (300 MHz, benzene-*d*$_6$) δ ppm 1.20 (s, 3 H), 1.12 (s, 6 H), 1.00 (s, 24 H); ^{13}C NMR (benzene-d$_6$,75MHz): δ ppm 83.5, 32.6, 25.3, 25.1; ^{11}B NMR (96 MHz, benzene-*d*$_6$) δ ppm 31.6 (s).

DippN(H)Bpin ^{1}H NMR (300 MHz, benzene-*d*$_6$) δ ppm 1.15 (s, 24 H) 1.30 (d, *J*=6.78 Hz, 24 H) 3.49 (m, *J*=6.88 Hz, 4 H) 7.10 - 7.22 (m, 3 H); ^{13}C NMR (benzene-d$_6$,75MHz): δ ppm 145.8, 136.4, 126.3, 123.6, 82.8, 29.0, 25.0, 24.2; ^{11}B NMR (96 MHz, benzene-*d*$_6$) δ ppm 27.5 (s).

Catalytic Reactions: ^{1}H and ^{11}B NMR spectra

Figure S9: ^{1}H NMR, Table 1, Entry 1: *n*-BuNH$_2$:HBpin

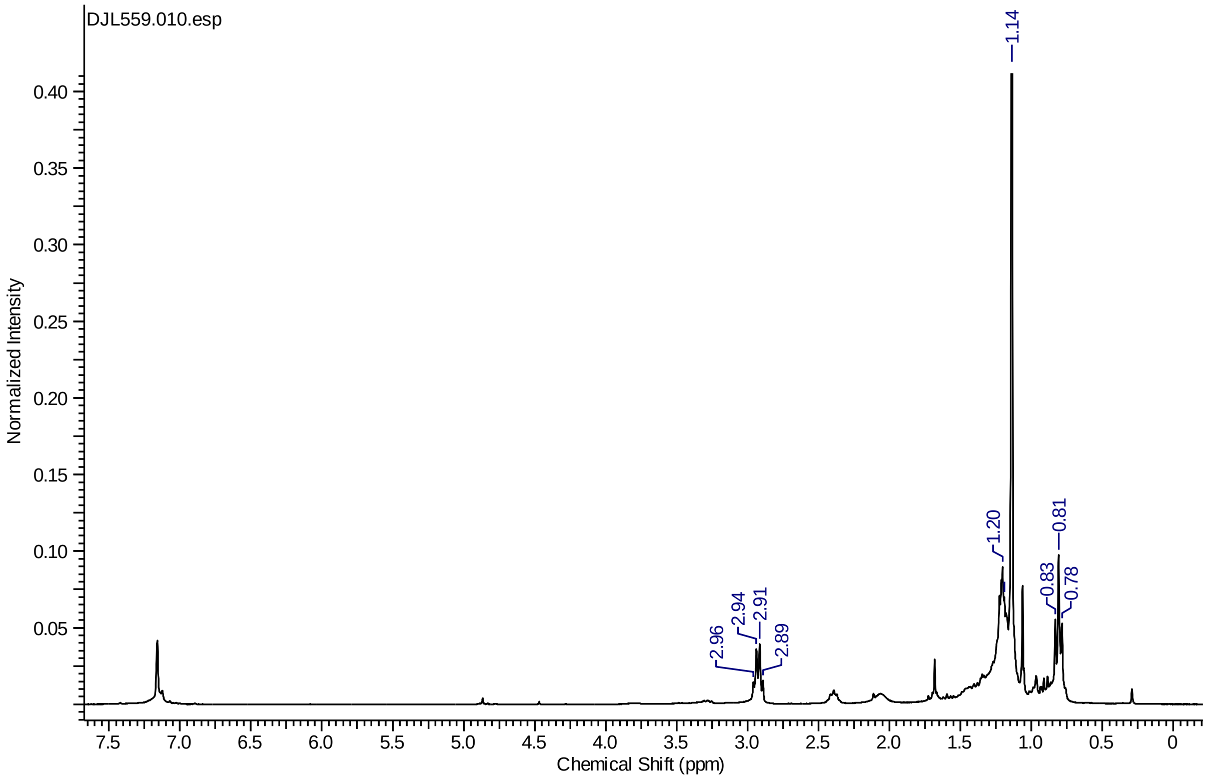

Figure S10: ^{11}B NMR, Table 1, Entry 1: *n*-BuNH$_2$:HBpin

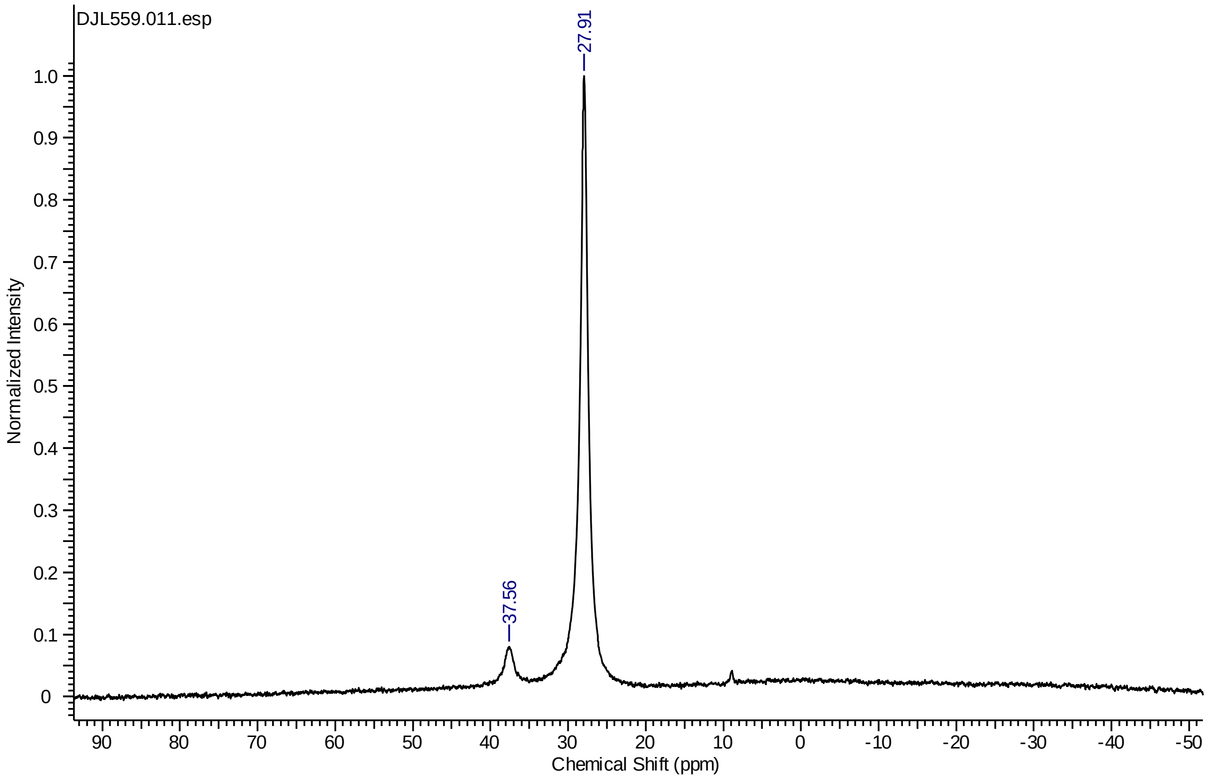

Figure S11: ^{1}H NMR, Table 1, entry 2: *t*-BuNH$_2$:HBpin

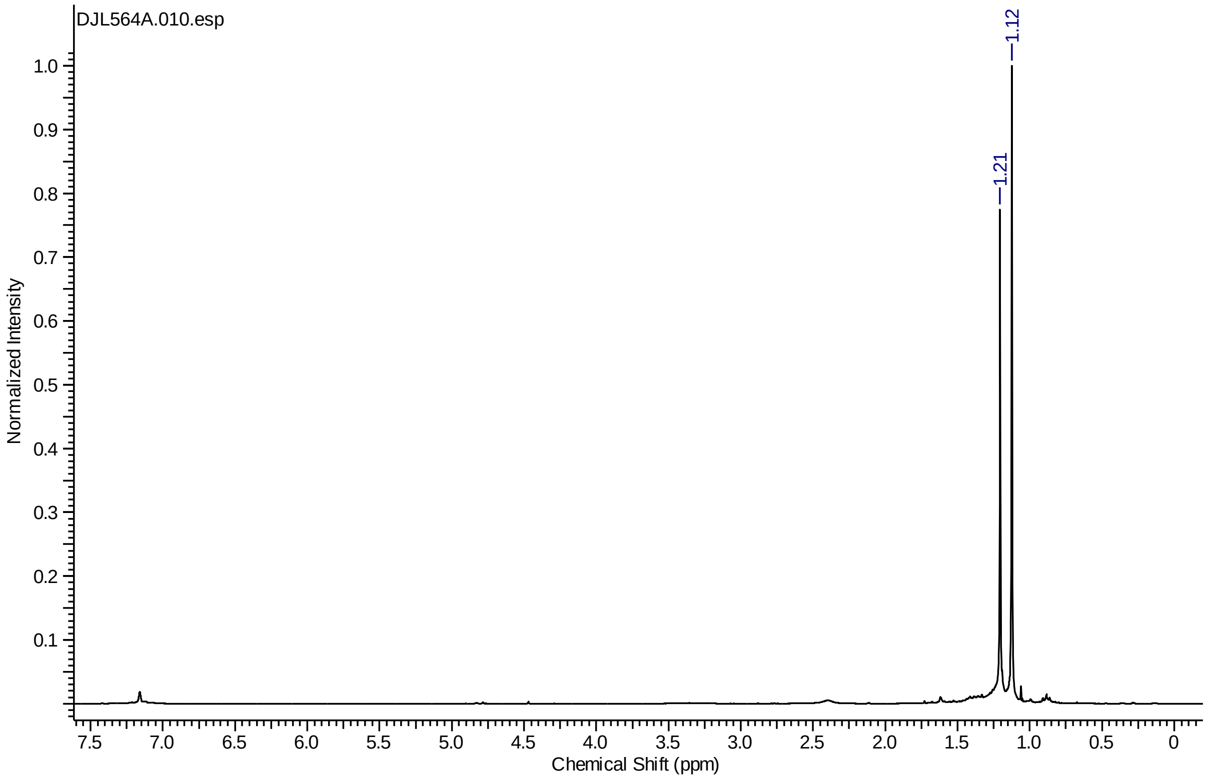

Figure S12: ^{11}B NMR, Table 1, entry 2: *t*-BuNH$_2$:HBpin

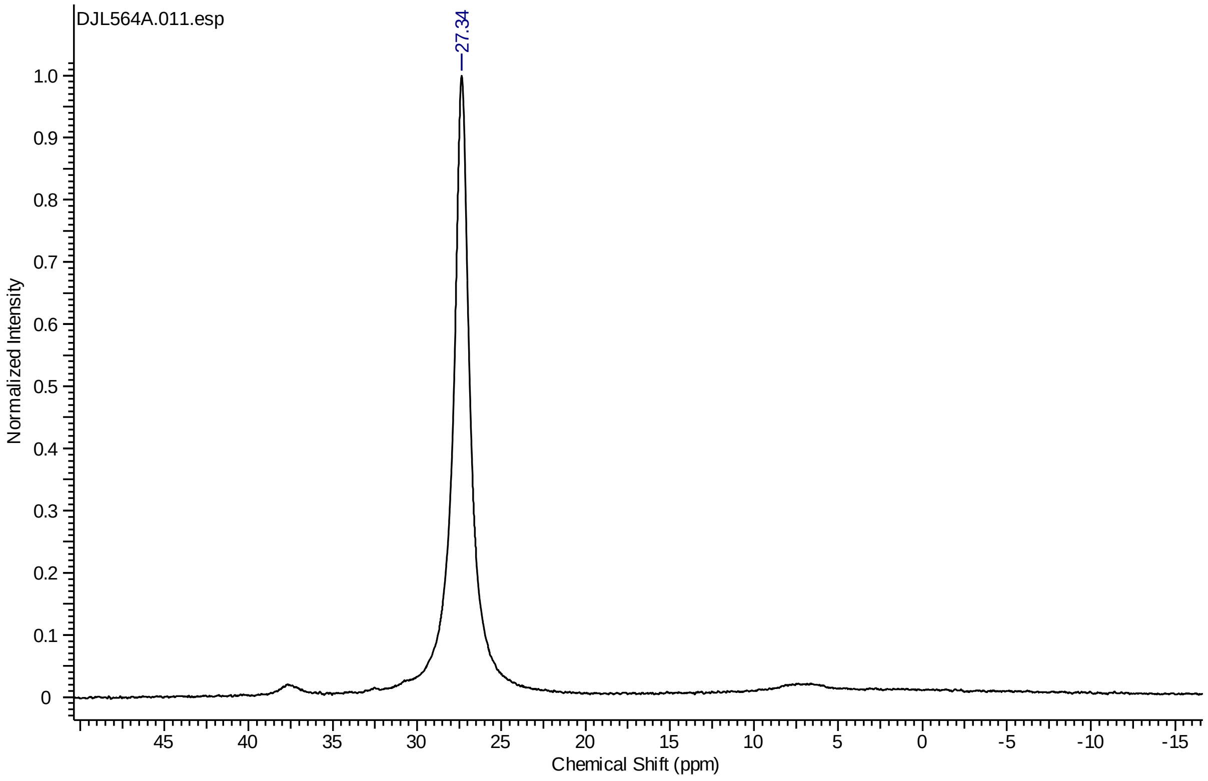

Figure S13: ^{1}H NMR, Table 1, entry 3: PhNH$_2$:HBpin

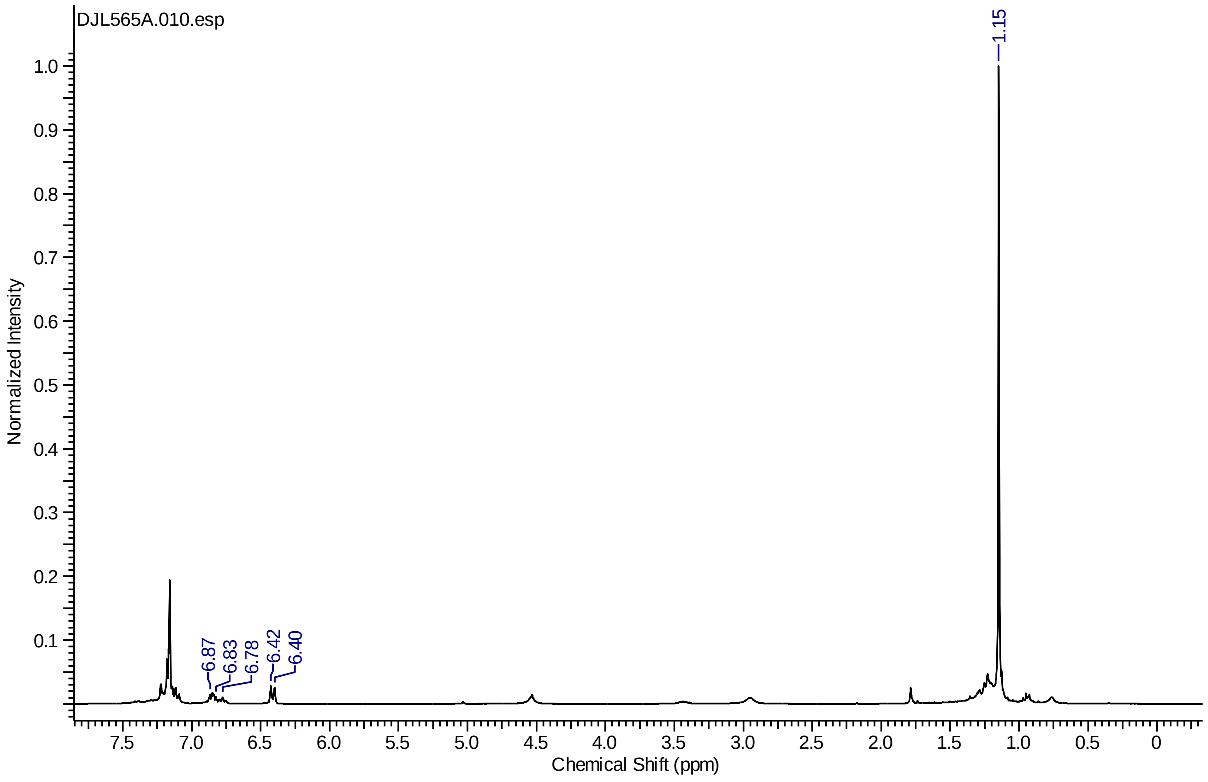

Figure S14: ^{11}B NMR, Table 1, entry 3: $PhNH_2$:HBpin

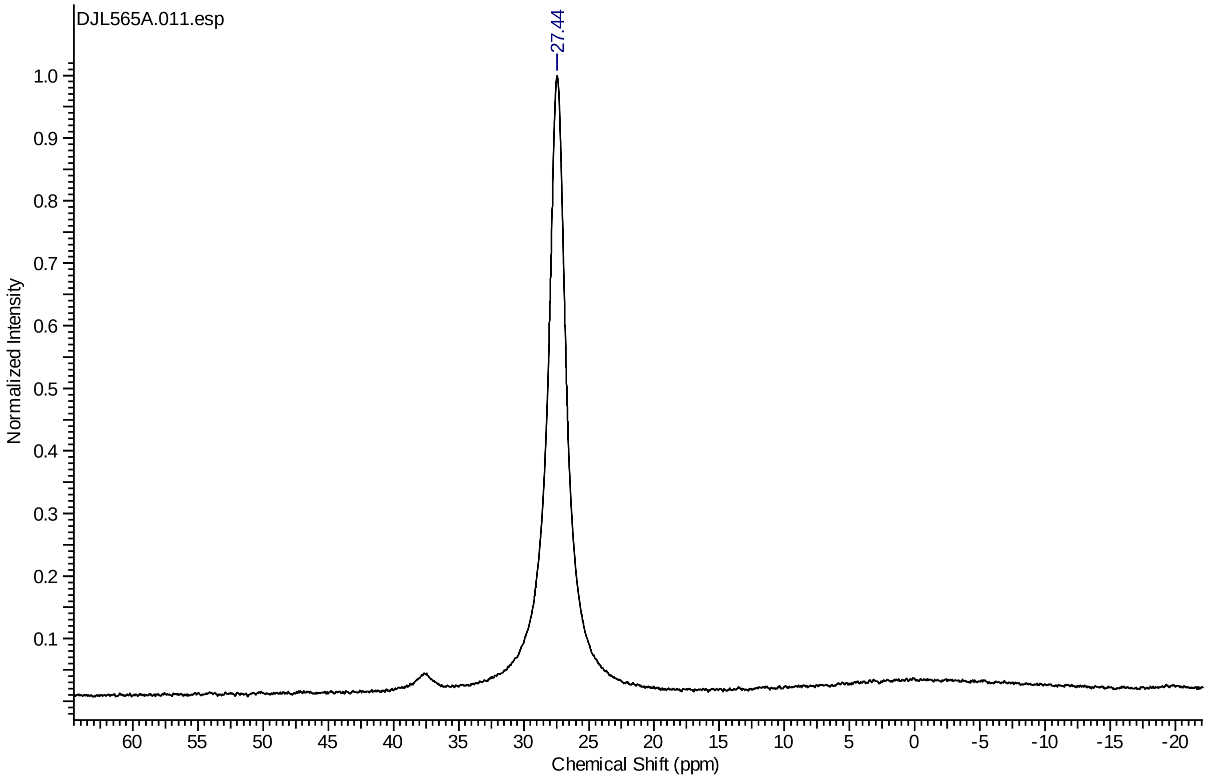

Figure S15: ^{1}H NMR, Table 1, entry 4: $DippNH_2$:HBpin

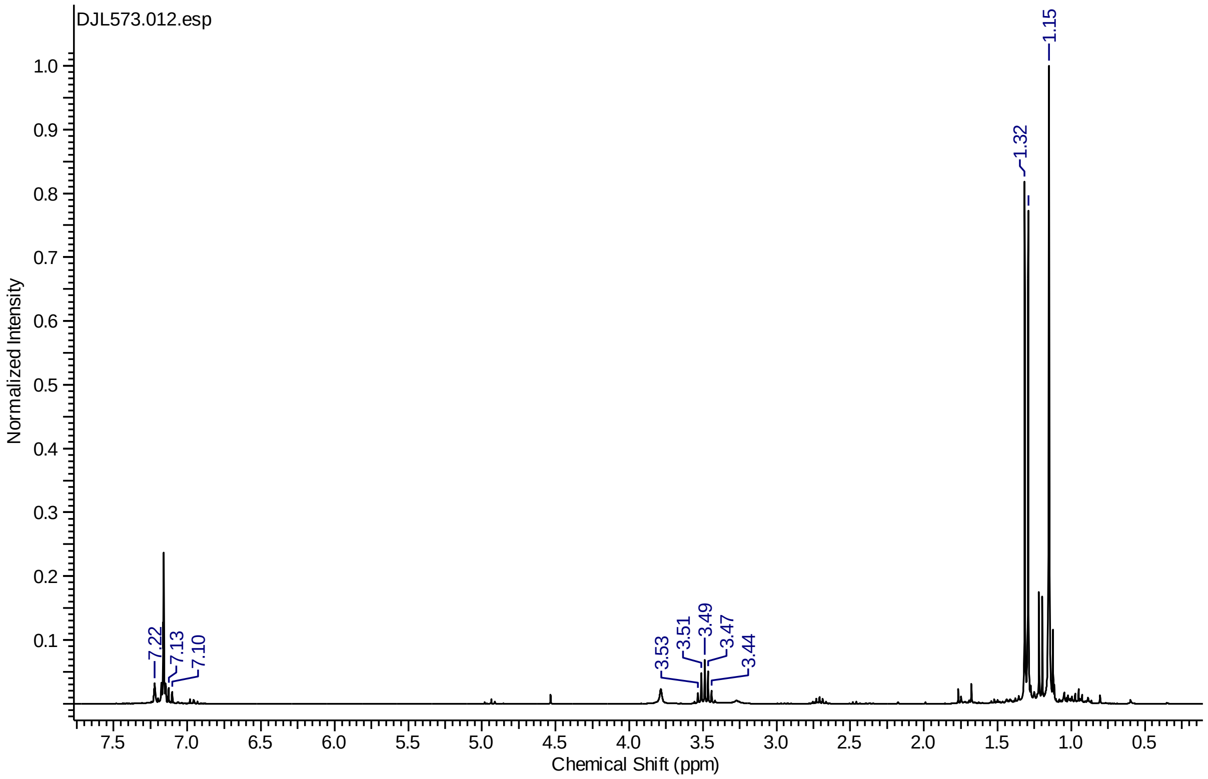

Figure S16: ^{11}B NMR, Table 1, entry 4: $DippNH_2$:HBpin

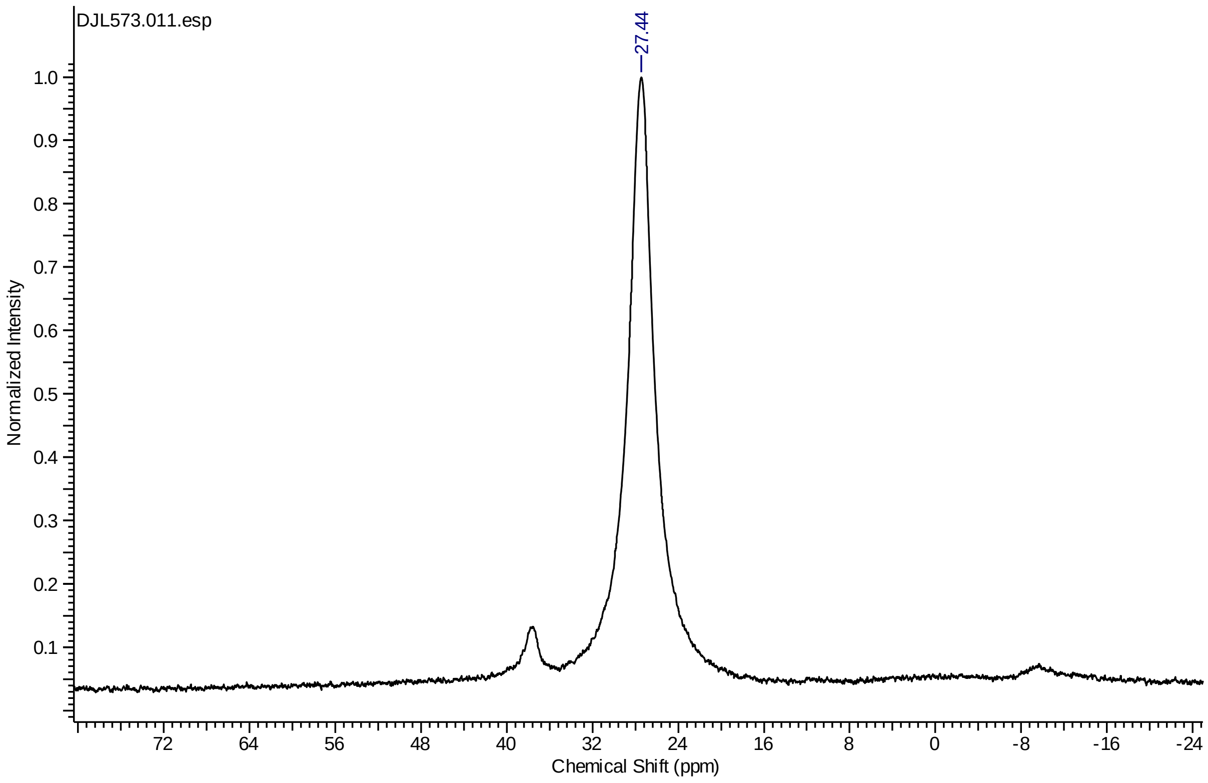

Figure S17: ^{1}H NMR, Table 1, entry 5: PhN(H)Me:HBpin

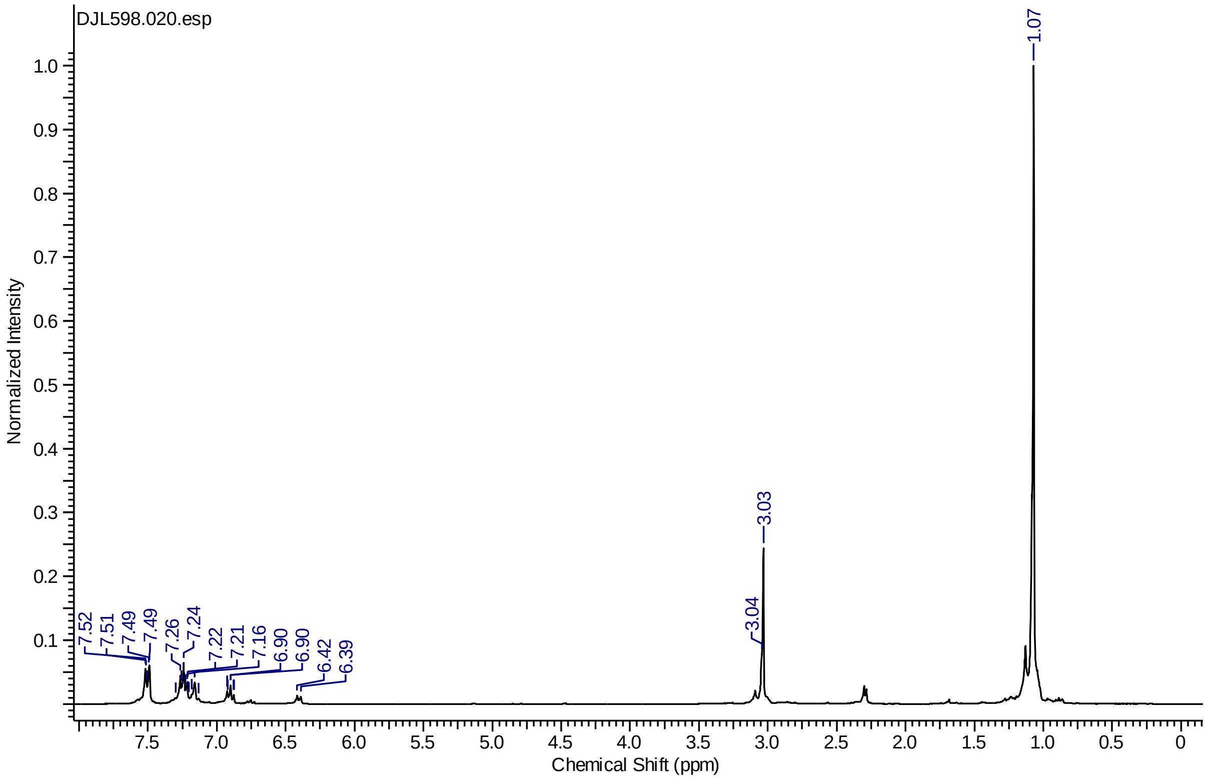

Figure S18: ^{11}B NMR, Table 1, entry 5: PhN(H)Me:HBpin

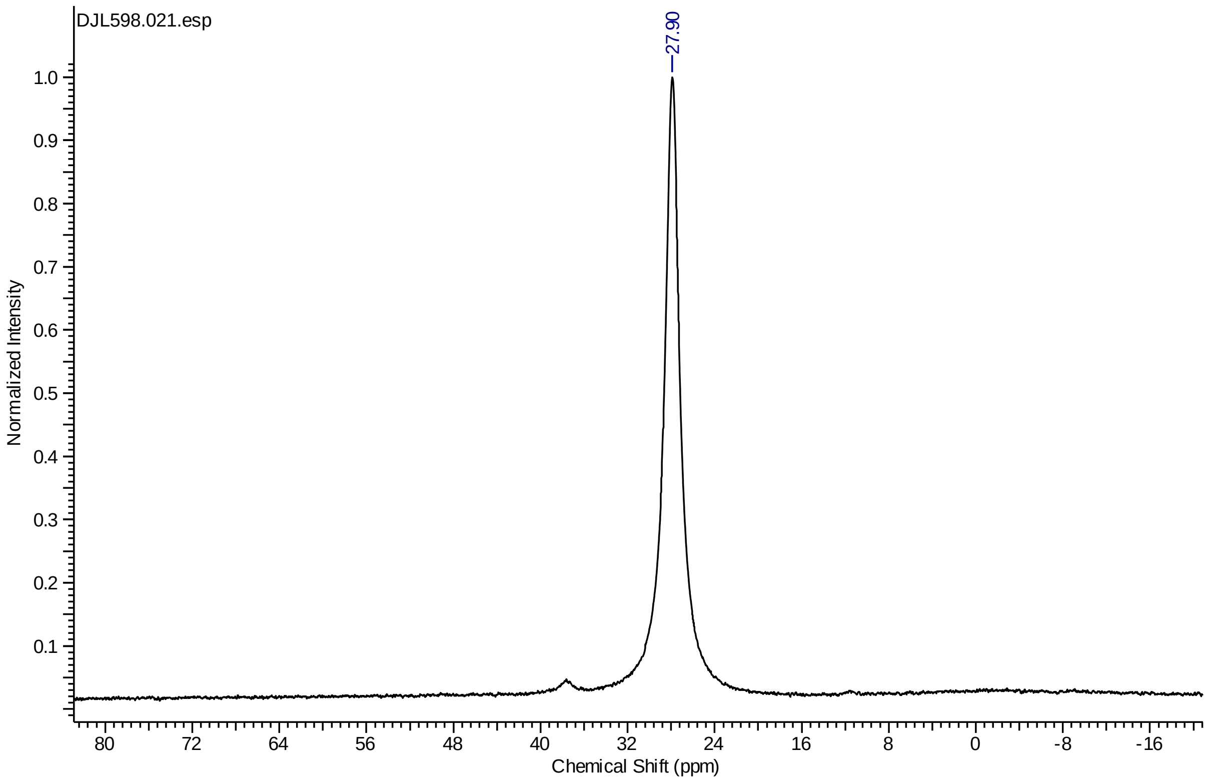

Figure S19: ^{1}H NMR, Table 1, entry 6: $(CH_2)_4$NH:HBpin

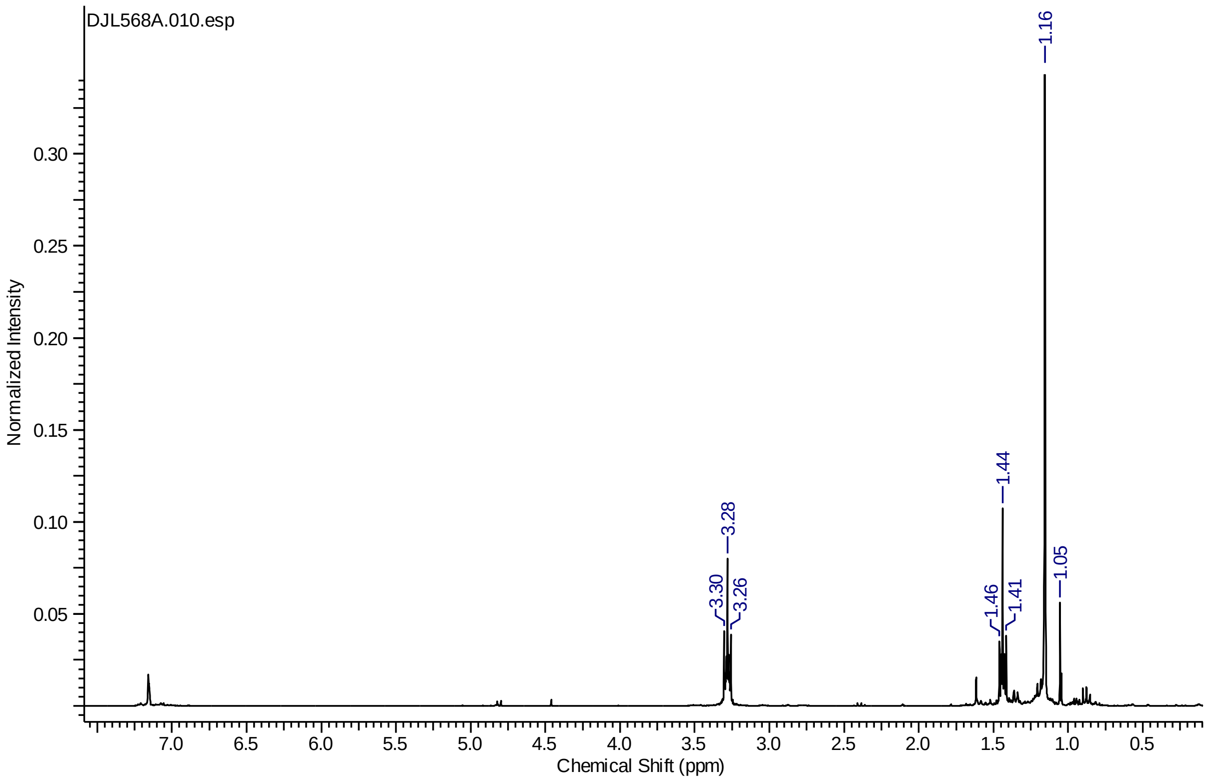

Figure S20: ^{11}B NMR, Table 1, entry 6: $(CH_2)_4NH$:HBpin

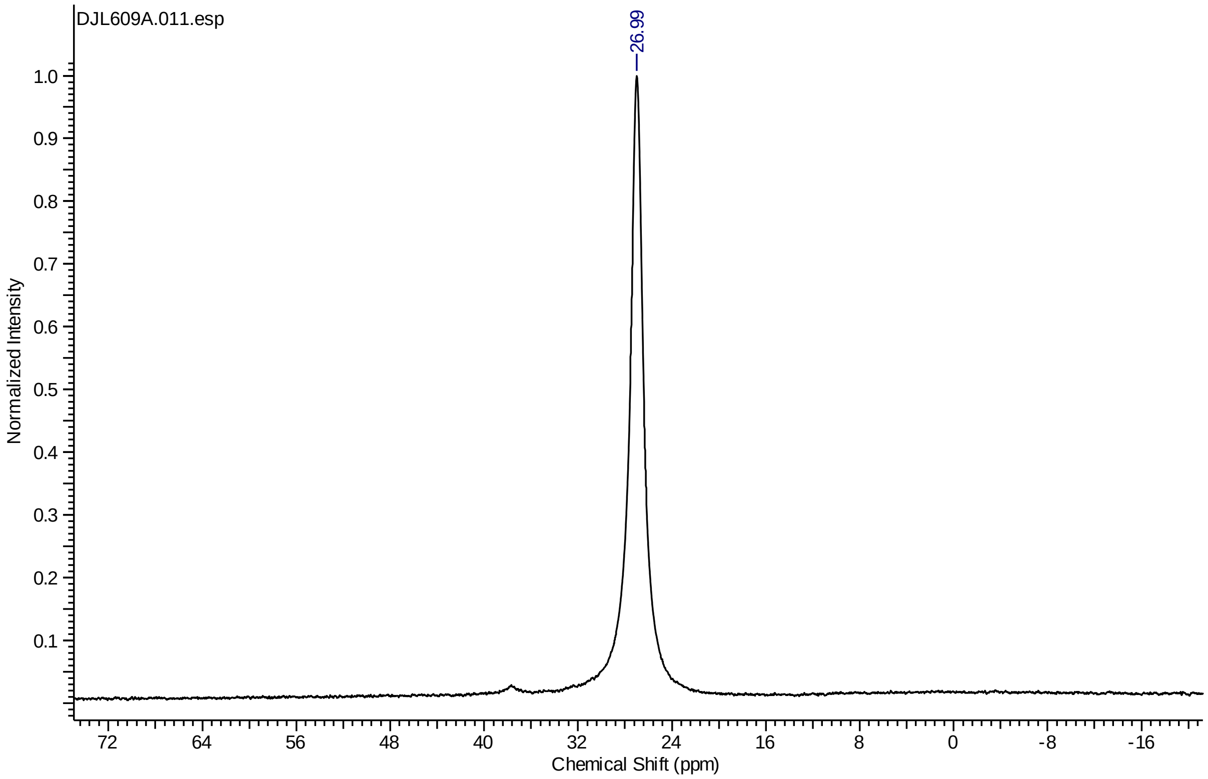

Figure S21: ^{1}H NMR, Table 1, entry 7: Et_2NH:HBpin

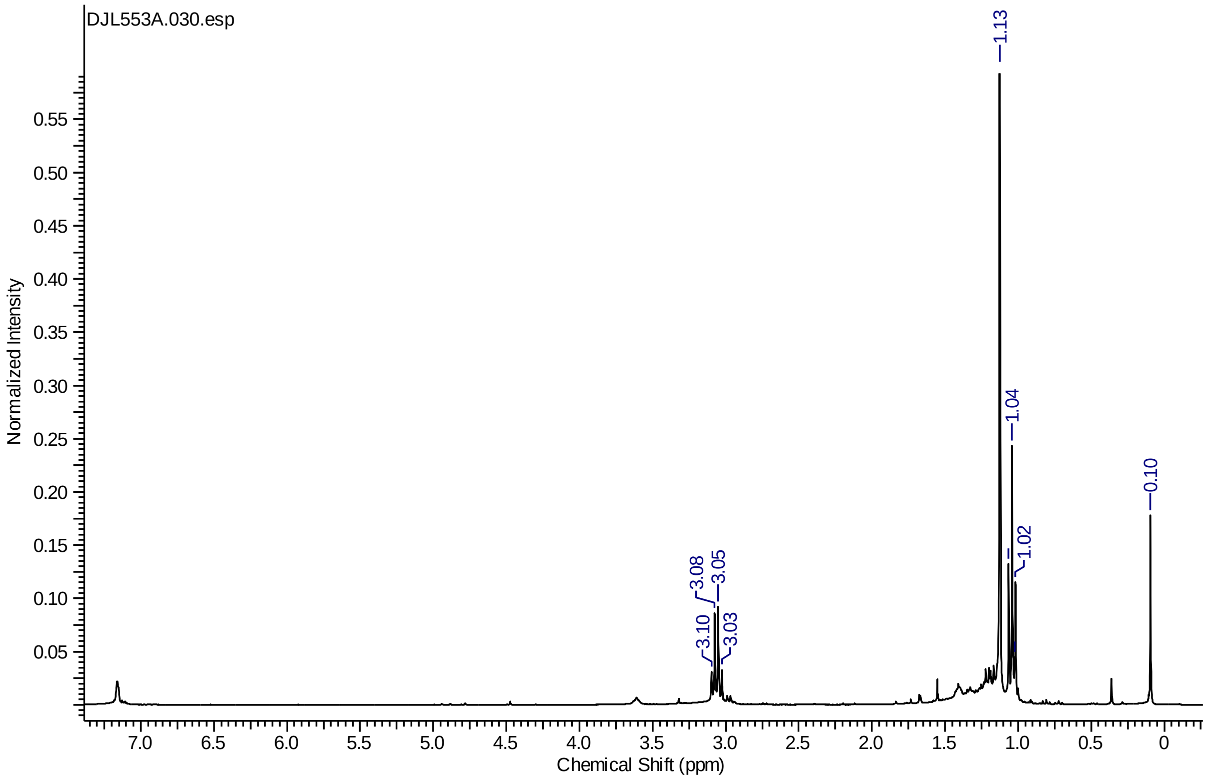

Figure S22: ^{11}B NMR, Table 1, entry 7: Et_2NH:HBpin

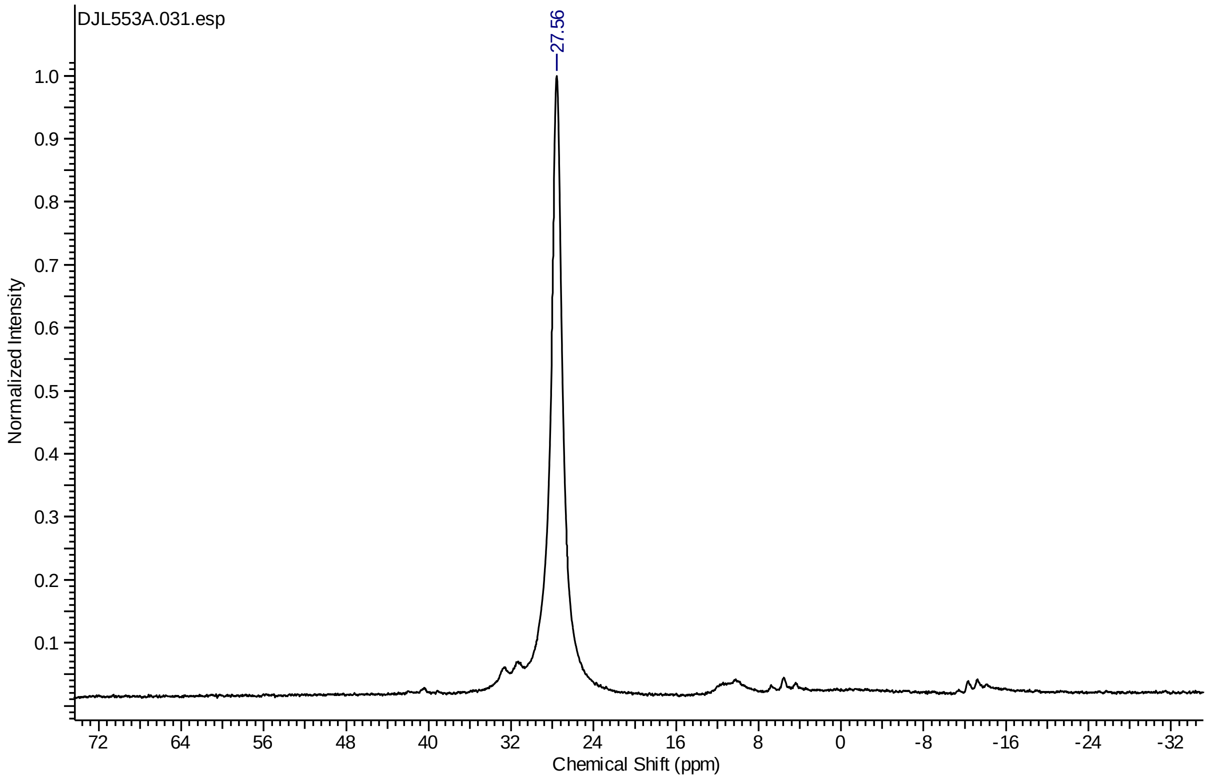

Figure S23: ^{1}H NMR, Table 1, entry 8: Ph_2NH:HBpin

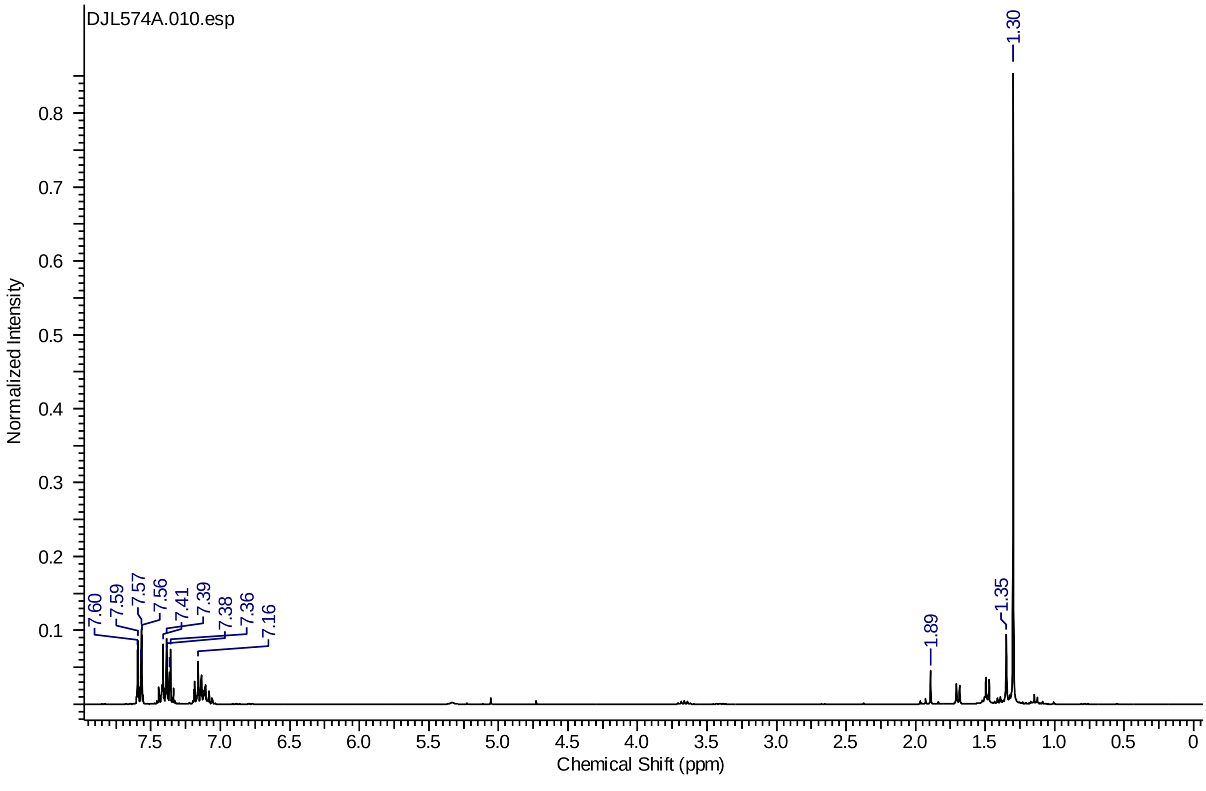

Figure S24: ^{11}B NMR, Table 1, entry 8: Ph_2NH:HBpin

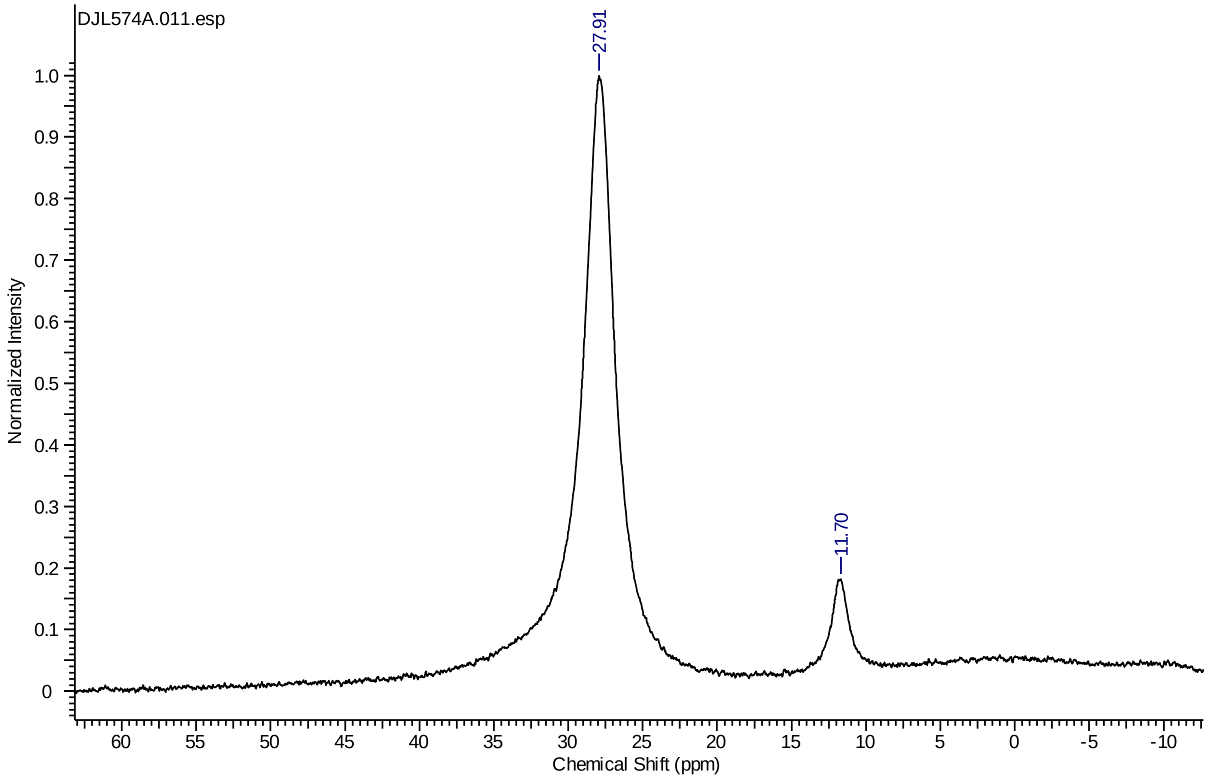

Figure S25: ^{1}H NMR, Table 1, entry 9: $(Me_3Si)_2NH$:HBpin, no reaction

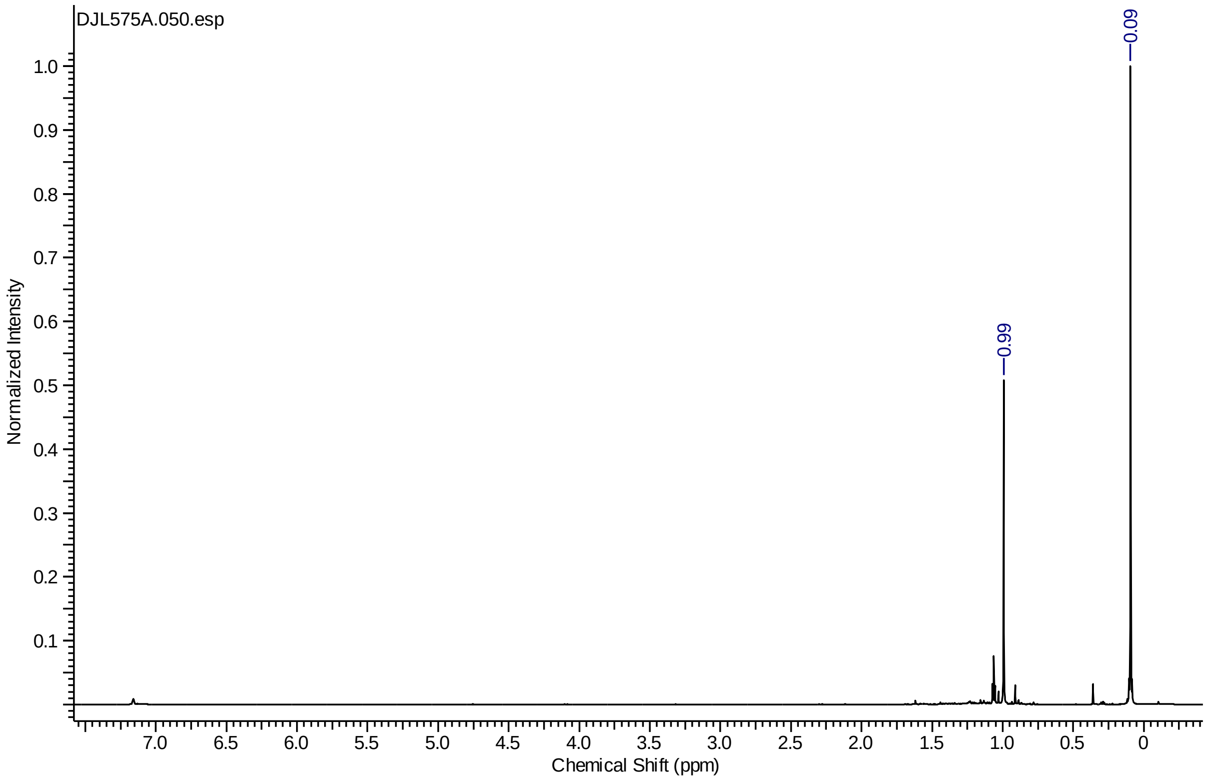

Figure S26: ^{11}B NMR, Table 1, entry 9: $(Me_3Si)_2NH$:HBpin, no reaction

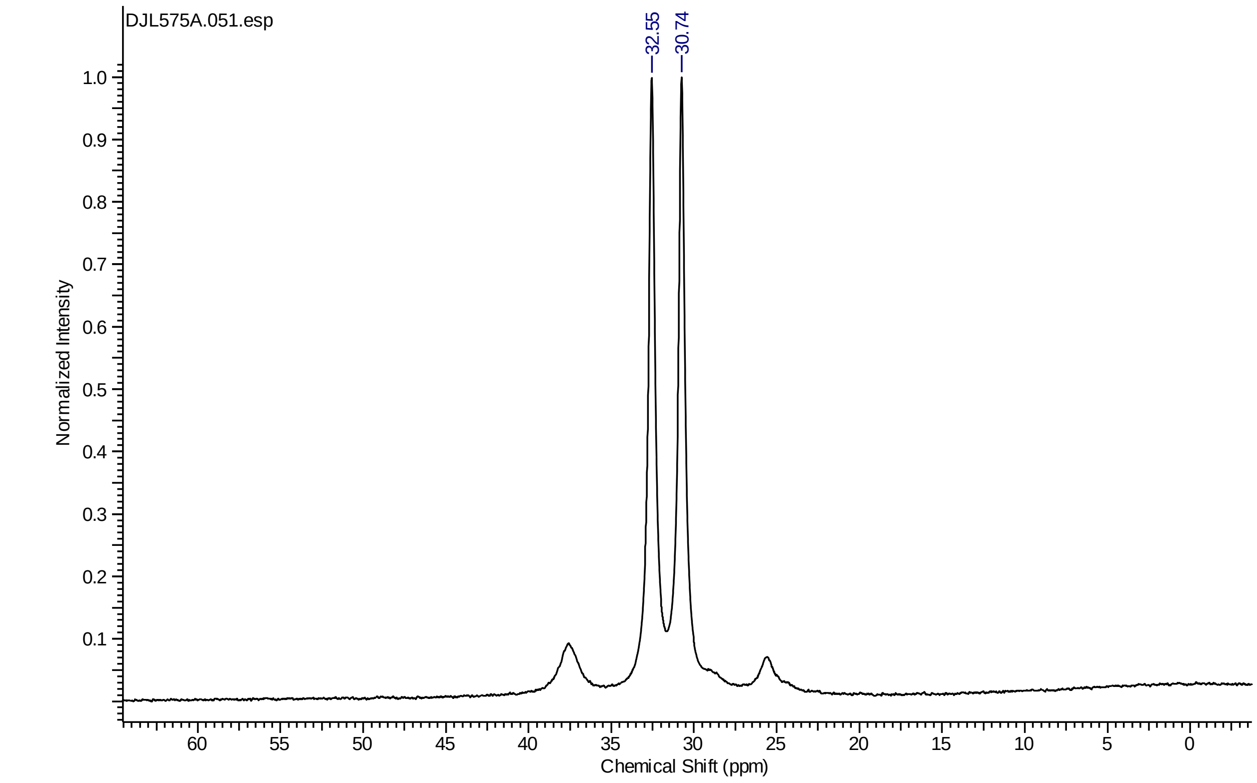

Figure S27: ^{1}H NMR, Table 1, entry 10: *n*-$BuNH_2$:9-BBN

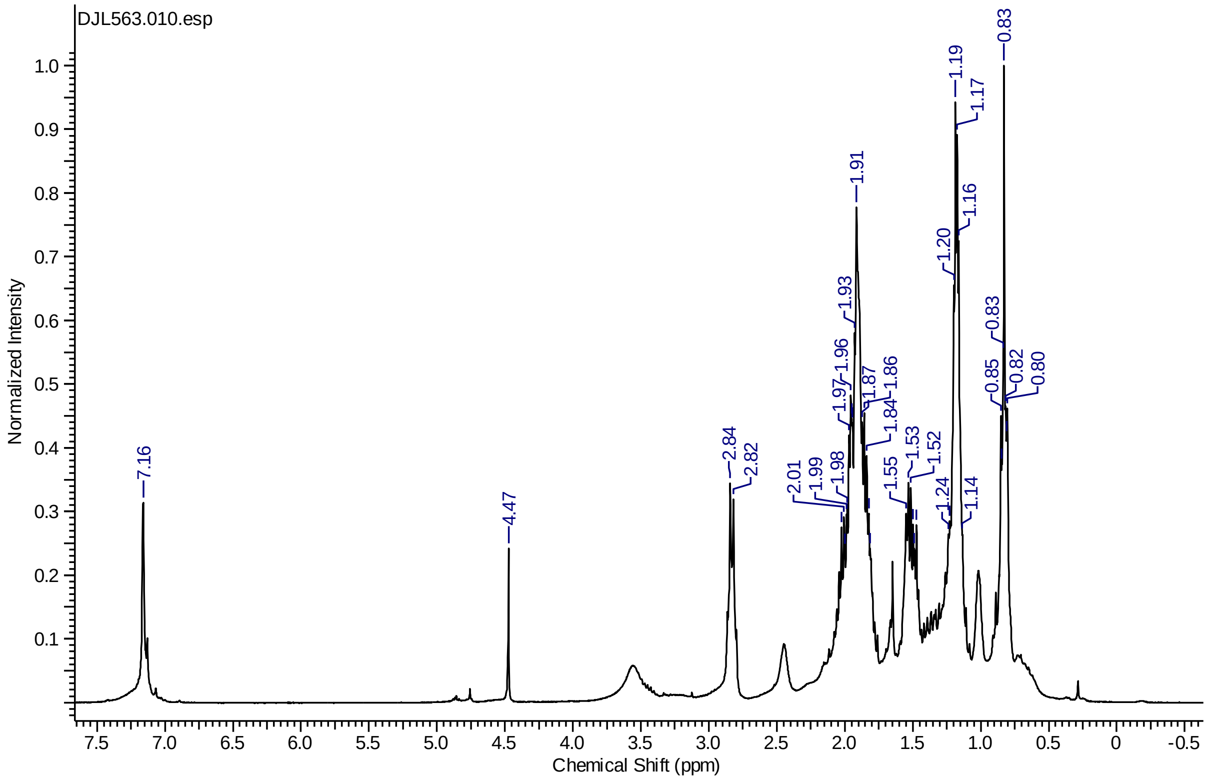

Figure S28: ^{11}B NMR, Table 1, entry 10: *n*-BuNH$_2$:9-BBN

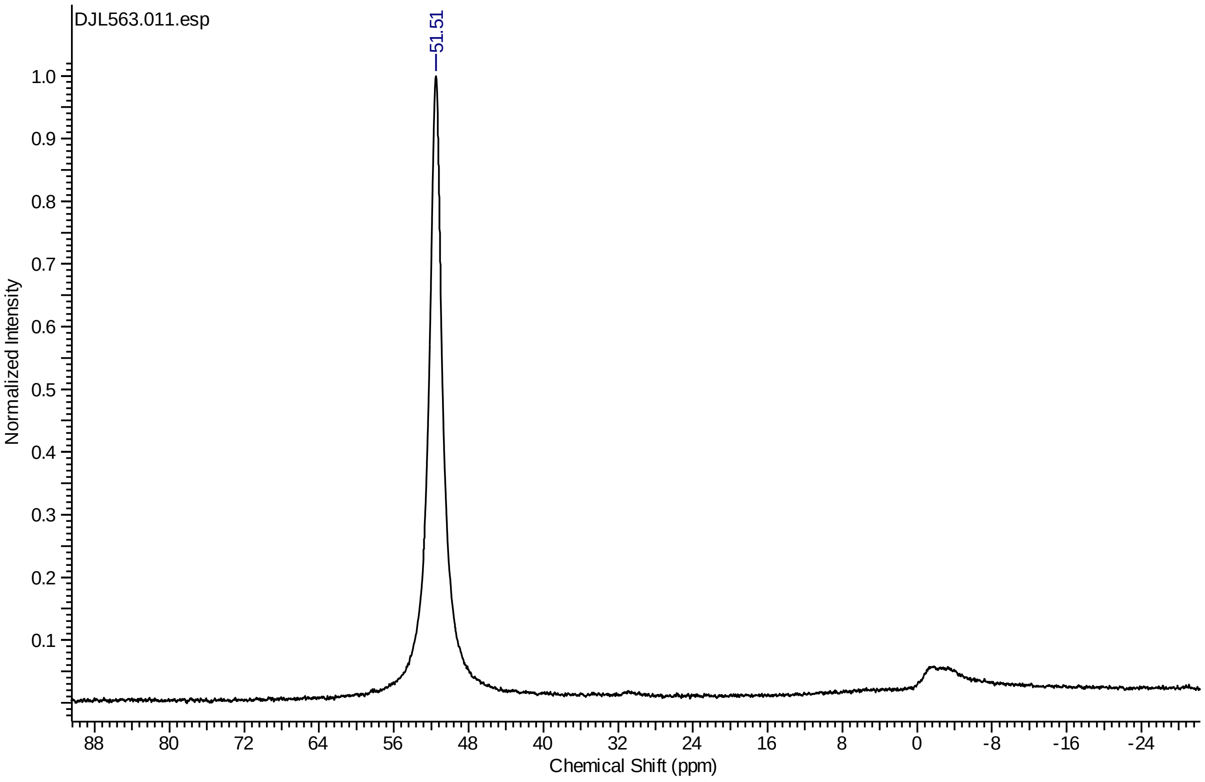

Figure S29: ^{1}H NMR, Table 1, entry 11: *t*-BuNH$_2$:9-BBN

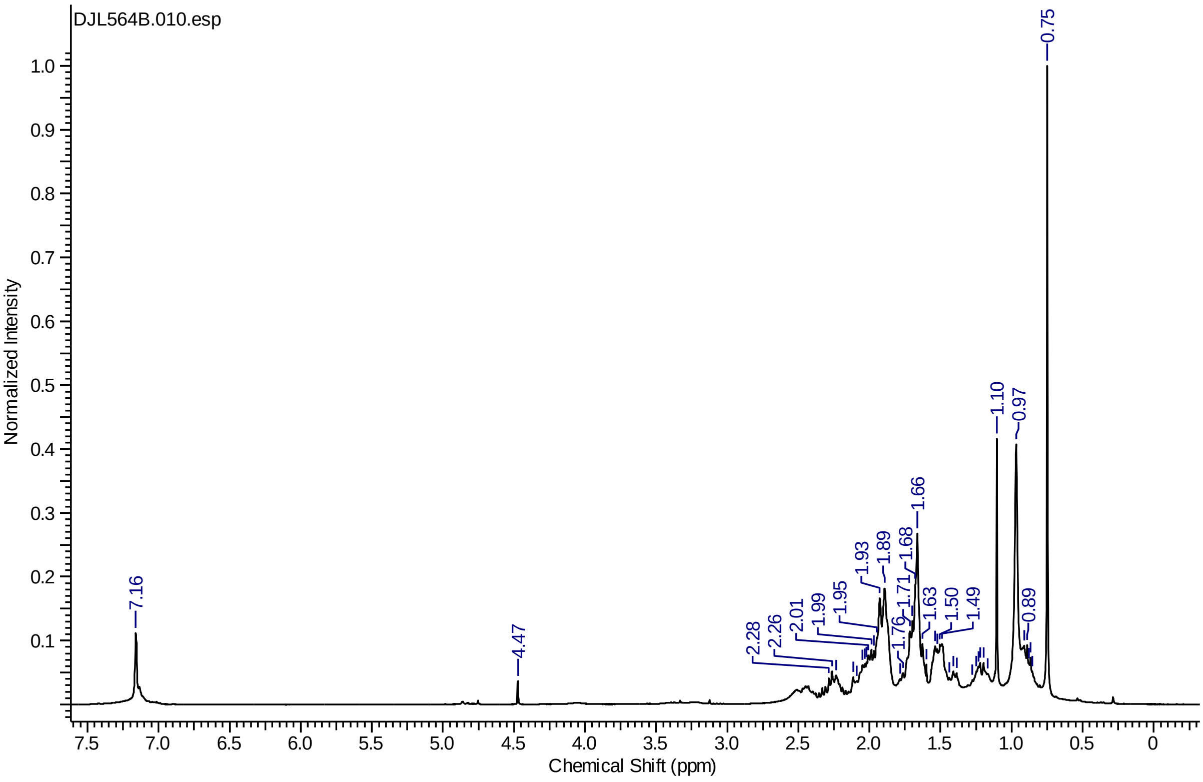

Figure S30: ^{11}B NMR, Table 1, entry 11: *t*-$BuNH_2$:9-BBN

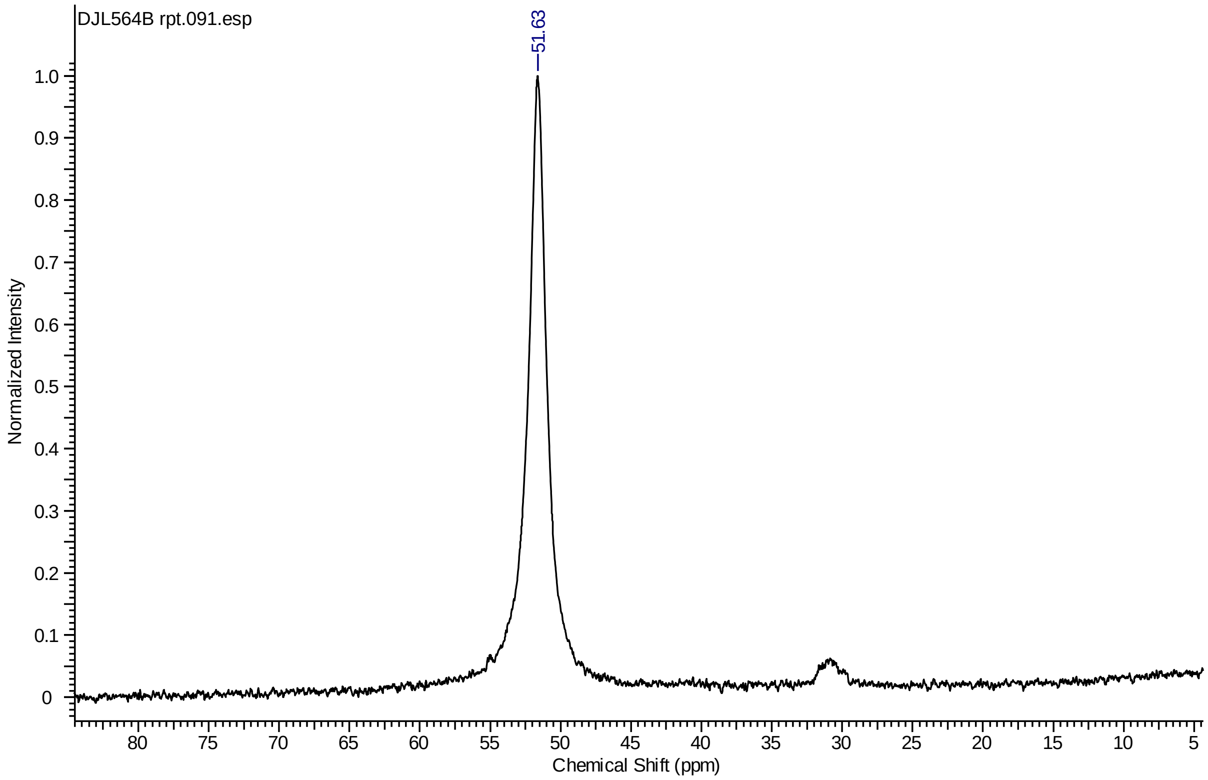

Figure S31: ^{1}H NMR, Table 1, entry 12: $PhNH_2$:9-BBN

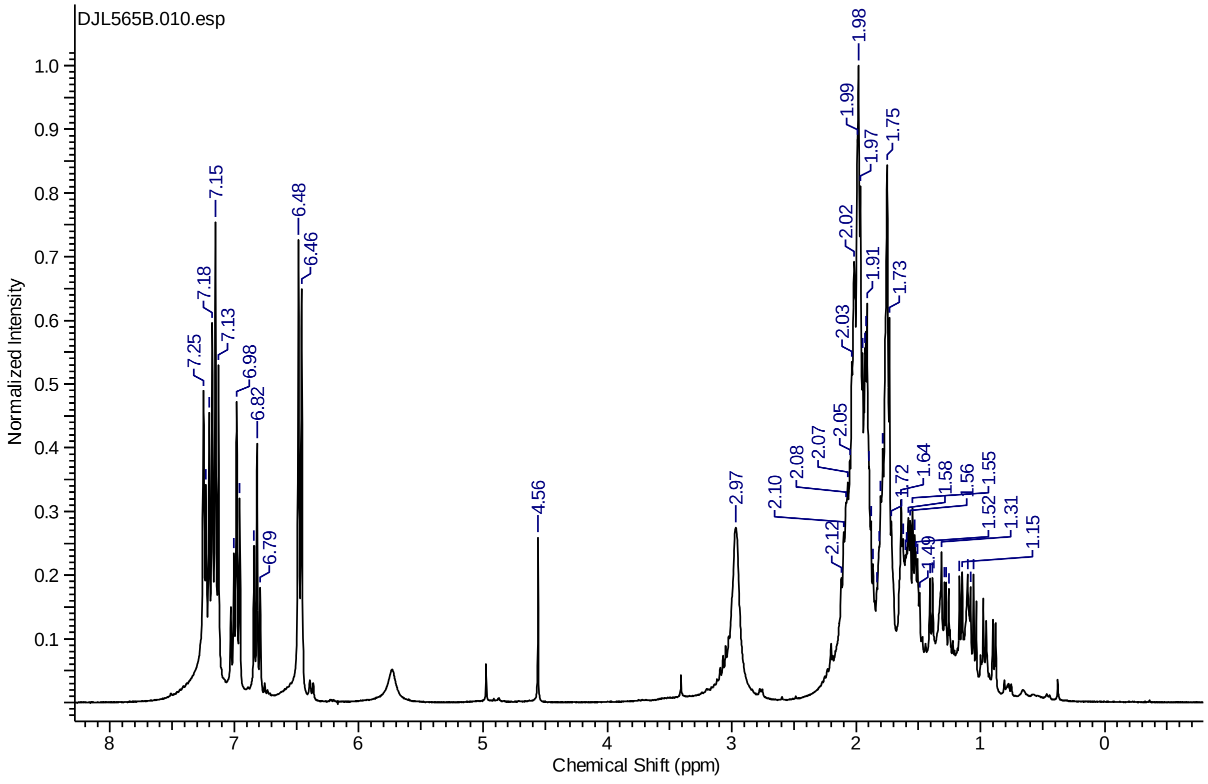

Figure S32: ^{11}B NMR, Table 1, entry 12: $PhNH_2$:9-BBN

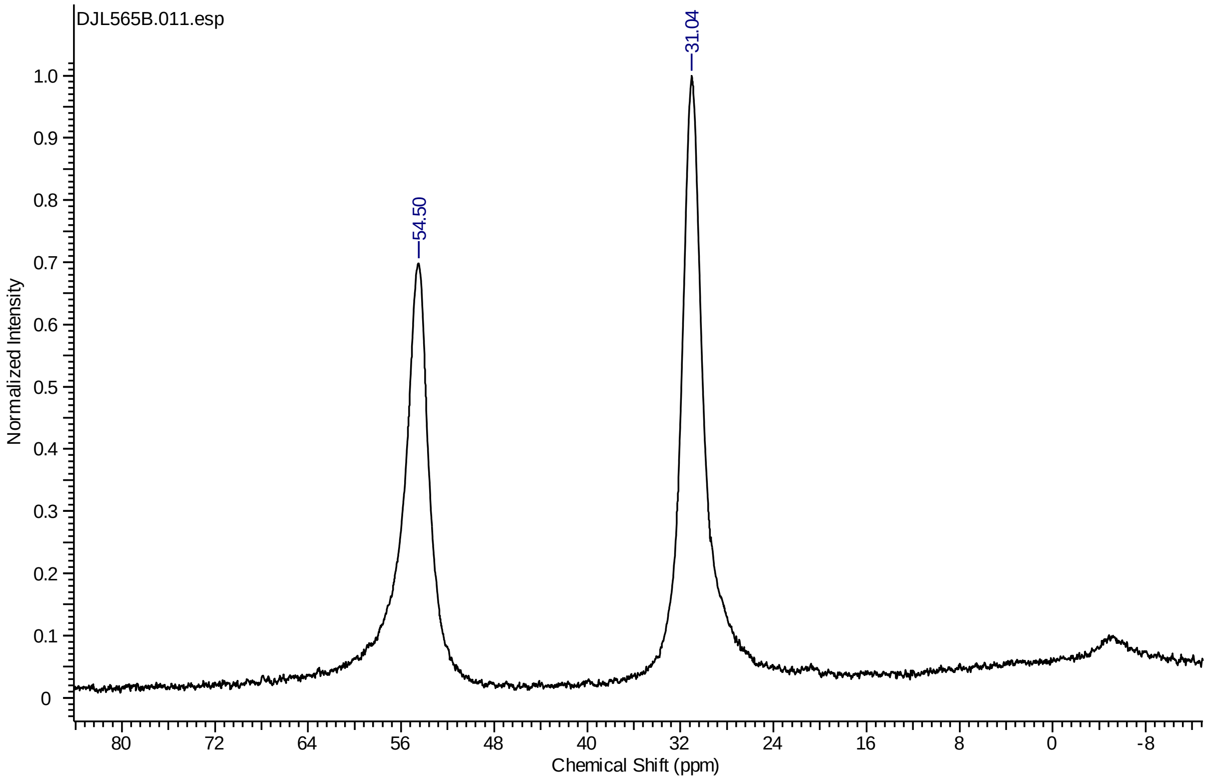

Figure S33: ^{1}H NMR, Table 1, entry 13: $DippNH_2$:9-BBN

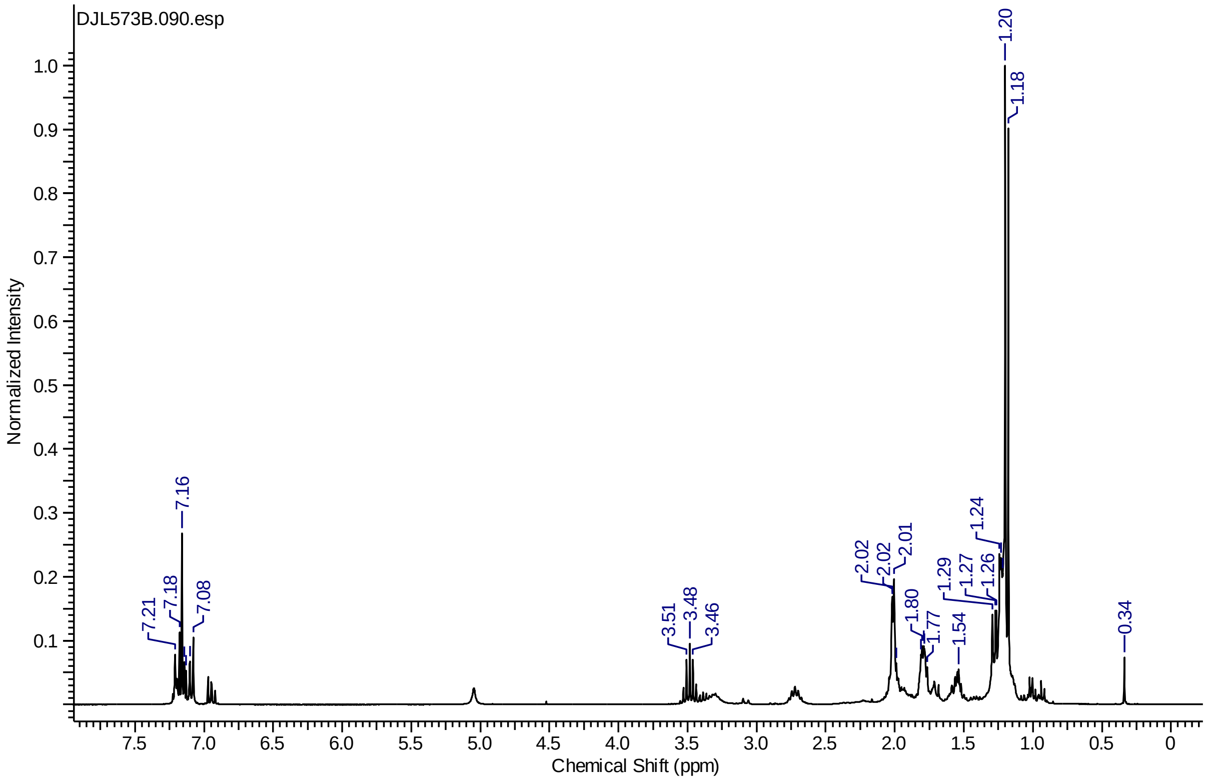

Figure S34: ^{11}B NMR, Table 1, entry 13: DippNH$_2$:9-BBN

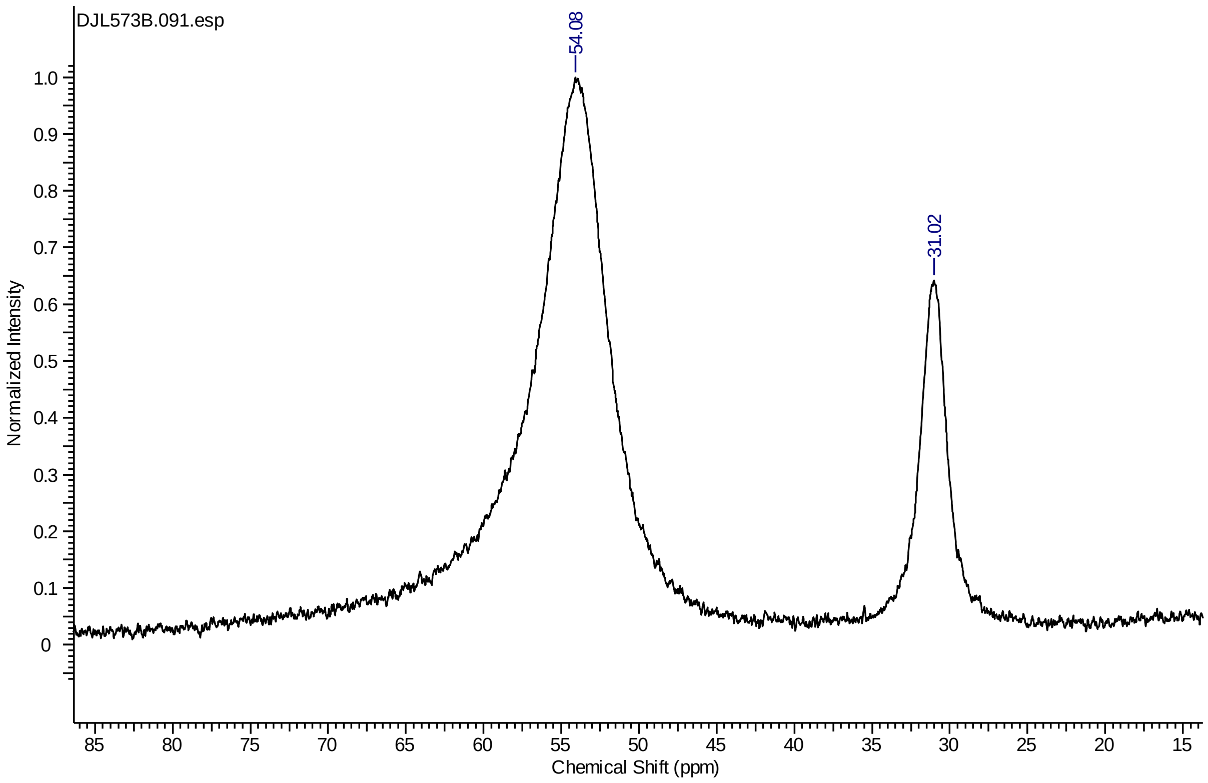

Figure S35: ^{1}H NMR, Table 1, entry 14: Ph$_2$NH:9-BBN

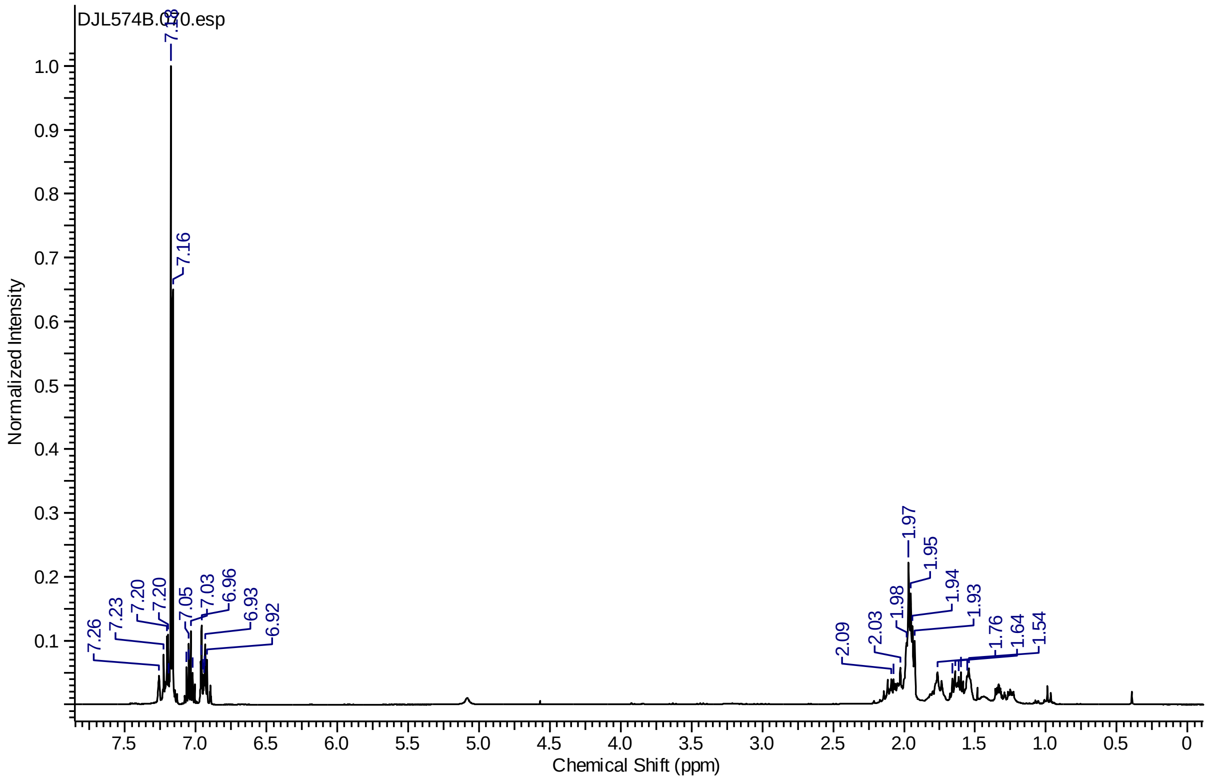

Figure S36: ^{11}B NMR, Table 1, entry 14: Ph_2NH:9-BBN

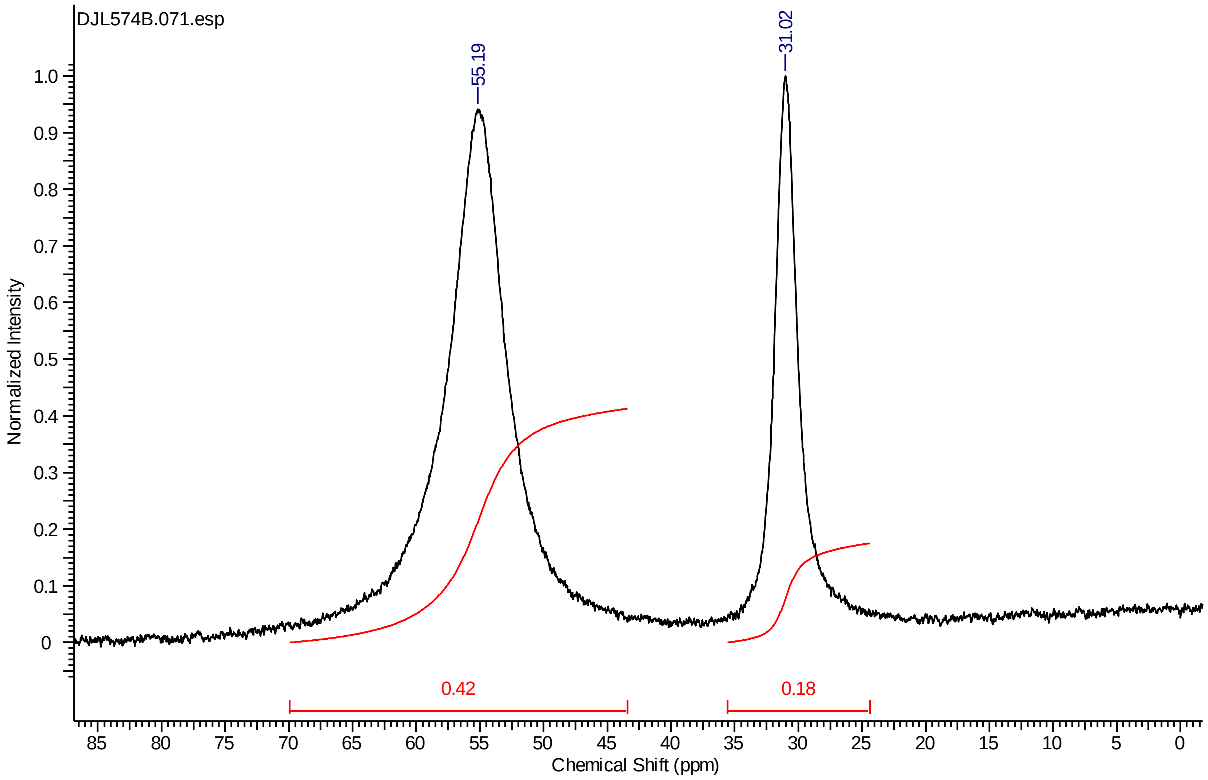

Figure S37: ^{1}H NMR, $(Me_3Si)_2NH$:9-BBN: no reaction

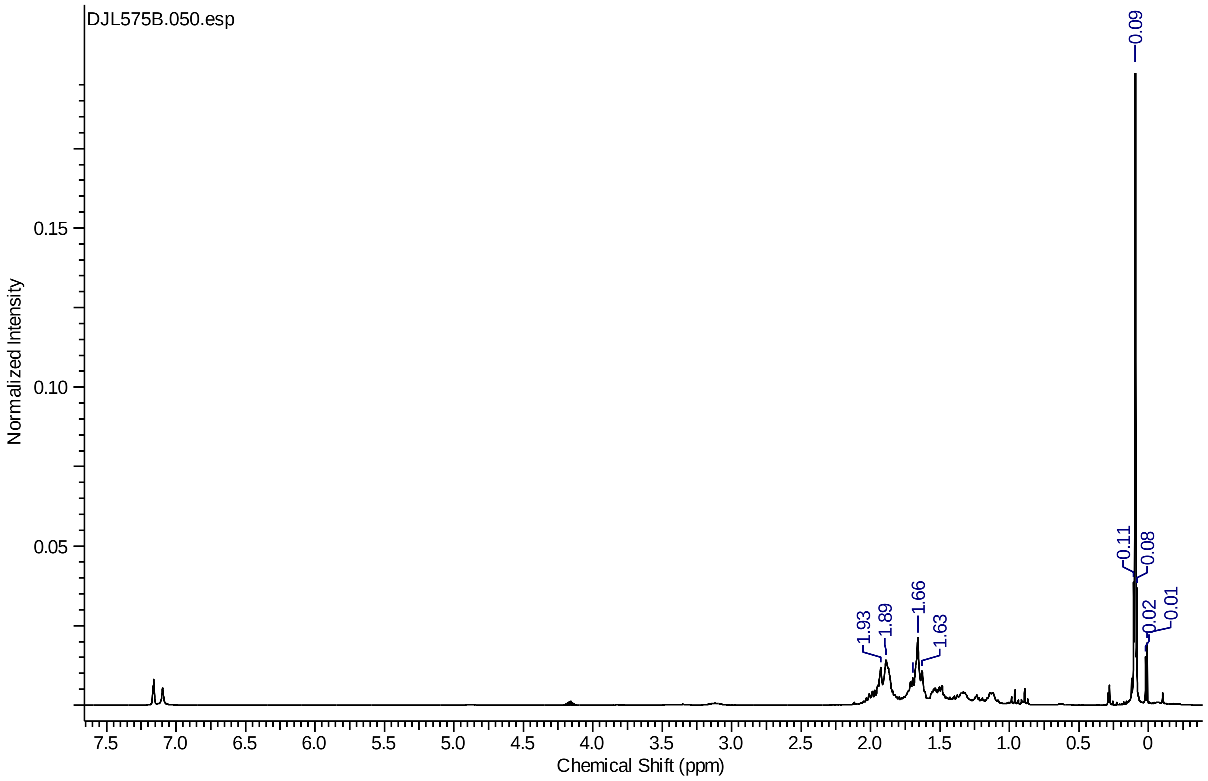

Figure S38: ^{11}B NMR, $(Me_3Si)_2NH$:9-BBN: no reaction

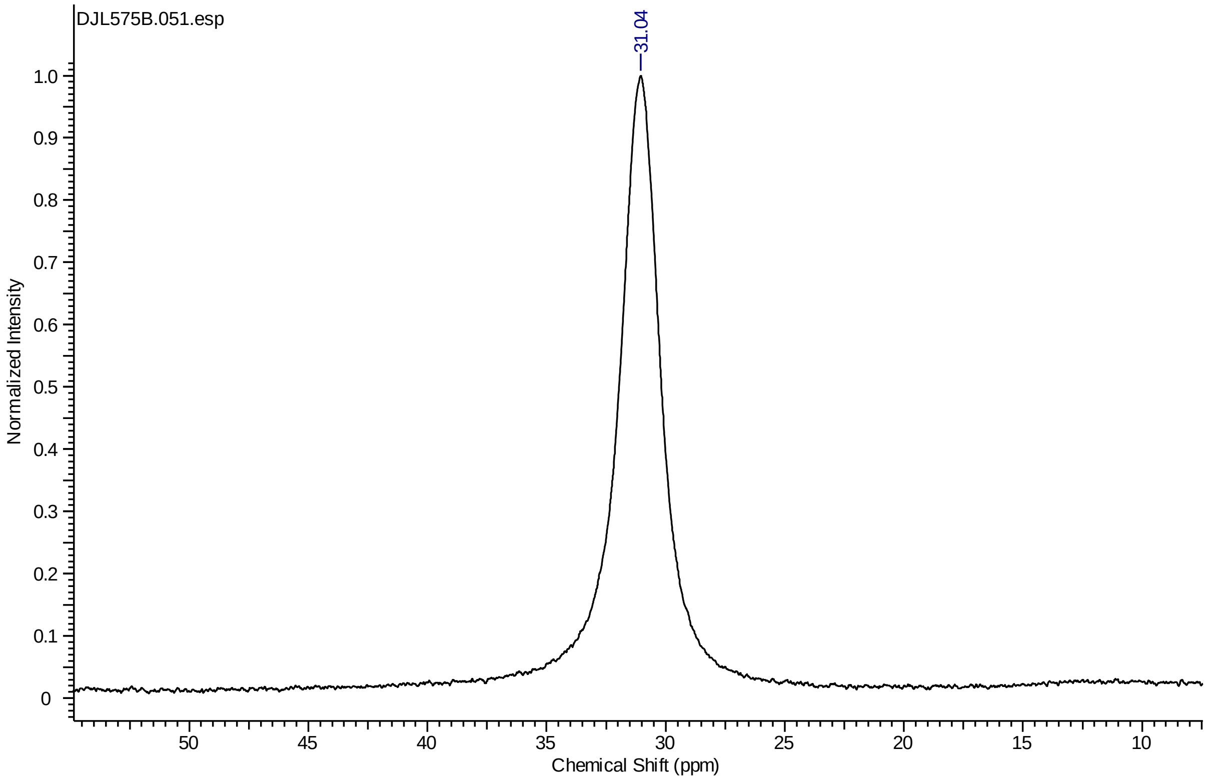

Kinetic Studies

In a glovebox a stock solution of the precatalyst (**I**) was made to the relevant concentration, 0.5 mL of the catalyst solution was transferred to a Youngs tap NMR tube followed by addition of the relevant quantity of borane, followed by the chosen amine substrate. The tube was sealed, removed from the glovebox, immediately frozen with liquid nitrogen and thawed just prior to loading into the NMR spectrometer which had been preheated to a chosen temperature (if required). ^{1}H NMR spectra were recorded at regular intervals. Reaction kinetics were monitored using the intensity changes in the substrate resonances over three or more half-lives on the basis of substrate consumption. Data were normalised against the initial substrate concentration $[\text{Substrate}]_{t=0}$ so that:

$$Ct = \frac{[Substrate]_{t=0}}{[Substrate]_{t=0} + [Substrate]_{t}}$$

Reaction rates were derived from the plot of Ct vs time (or Ln(Ct), 1/Ct) by using linear trendlines generated by Microsoft Excel software. To obtain Arrhenius and Eyring plots, kinetic analyses were conducted at 4-5 different temperatures, each separated by approximately 5 K.

Figure S39: Representative kinetic ^{1}H NMR spectra for the reaction of PhN(H)Me and HBpin catalysed by 5 mol% **I** and recorded every 30 minutes at 298 K.

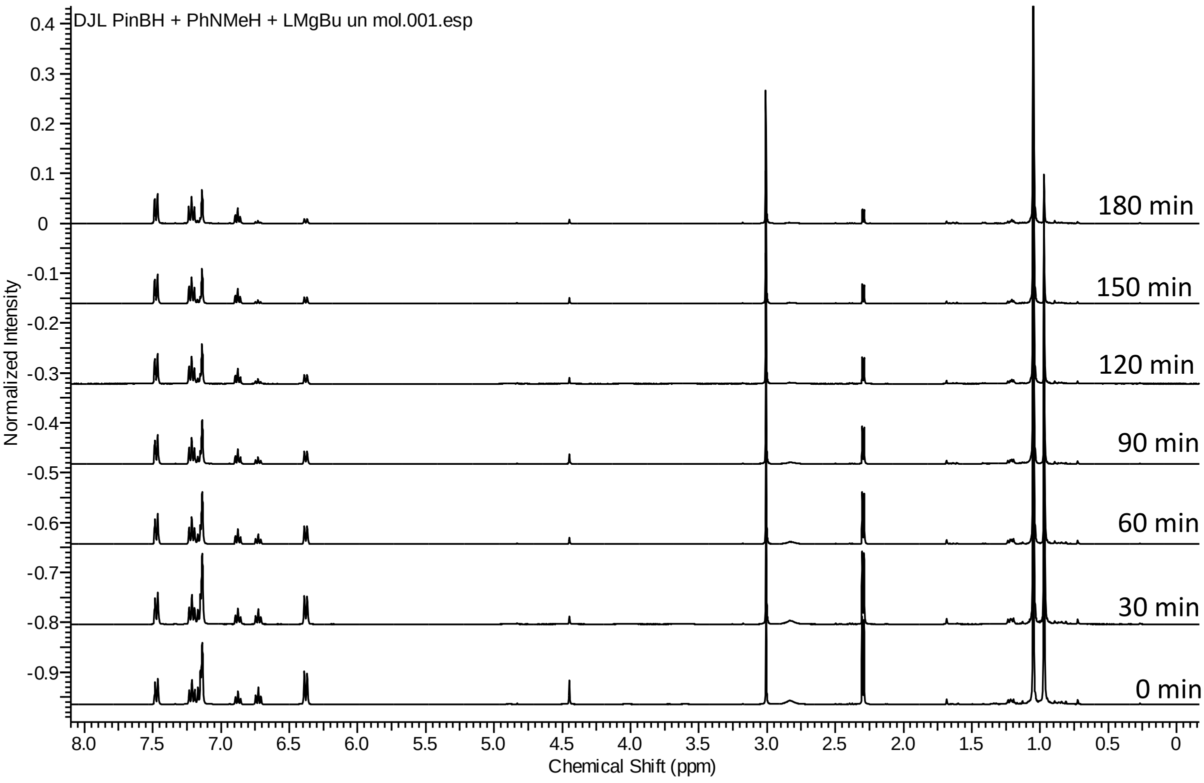

Kinetic data for the dehydrocoupling of PhNH(Me) coupling and 9-BBN catalyzed by I

Figure S40: The overall first-order plots of ln([amine]$_t$/[amine]$_0$) against time for a range of **I** concentrations.

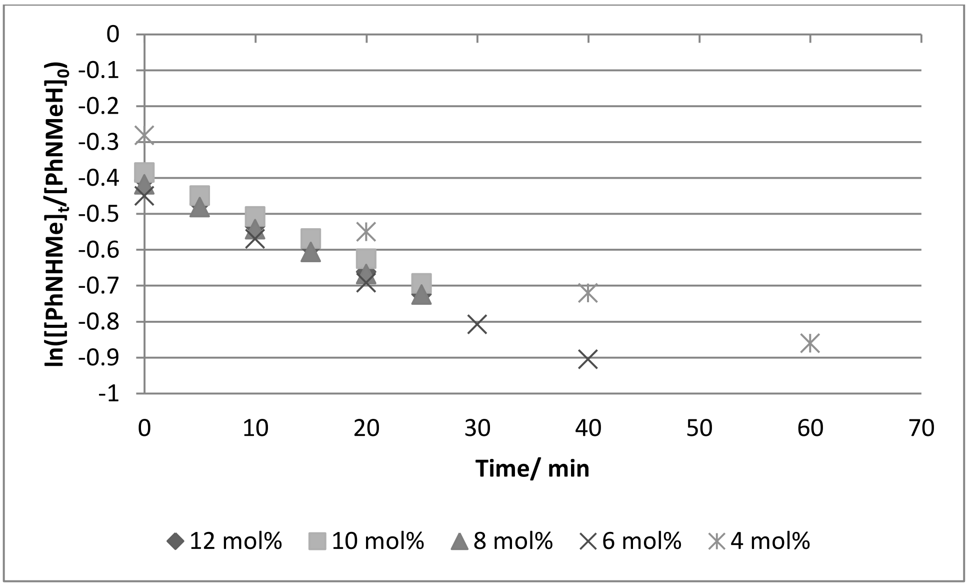

Figure S41: The plot of observed rate constant (k_{obs}) against $[\mathbf{I}]^{1/2}$ for a range of [**I**].

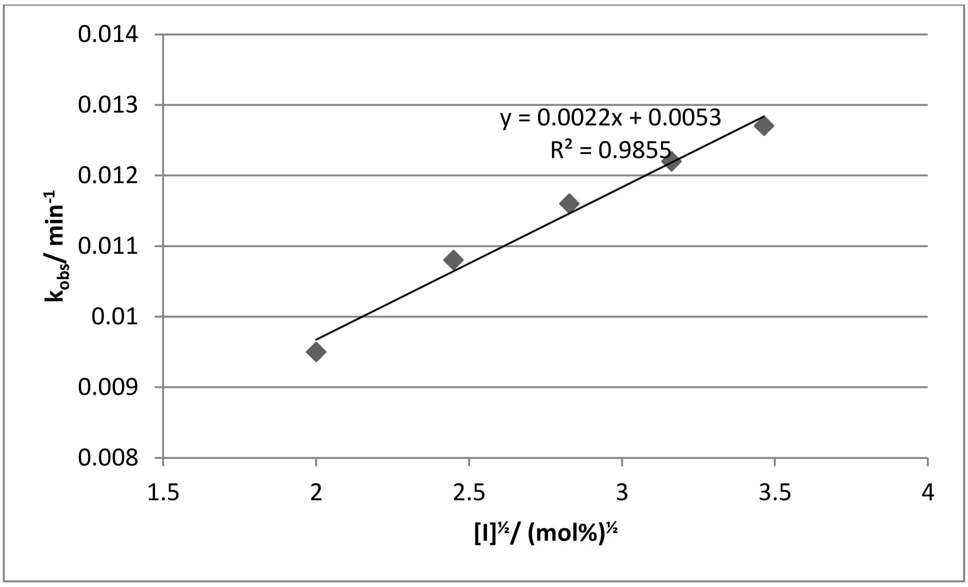

Figure 42: The overall best fit inverse-order plot of f([borane]$_t$/[borane]$_0$) against time for a reaction with a tenfold excess of *N*-methylaniline.

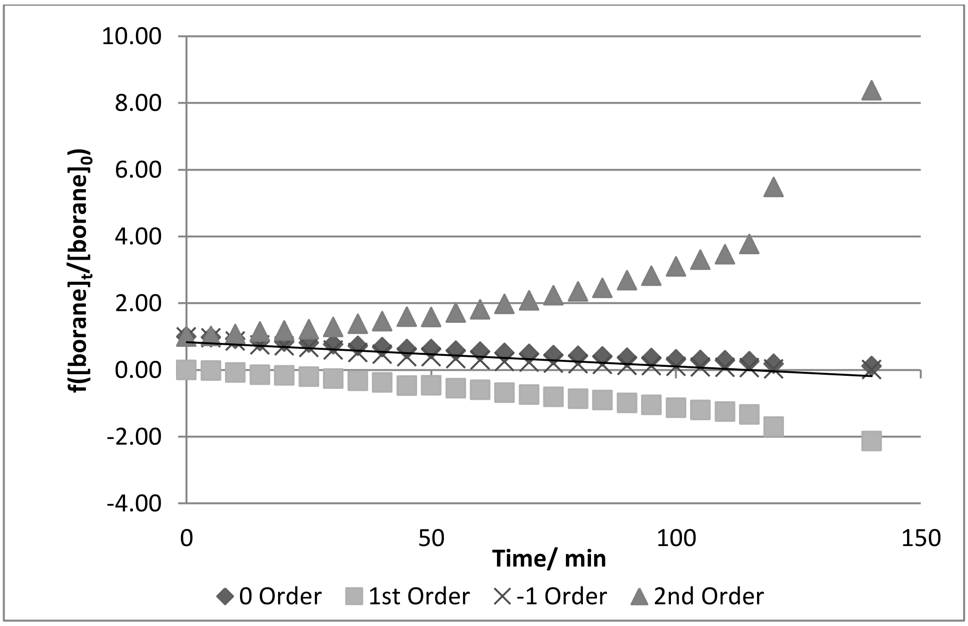

Kinetic data for the dehydrocoupling of PhNH(Me) coupling and HBpin catalyzed by I

Figure S43: The overall first-order rate plots of ln([amine]$_t$/[amine]$_0$) against time for a range of **I** concentrations.

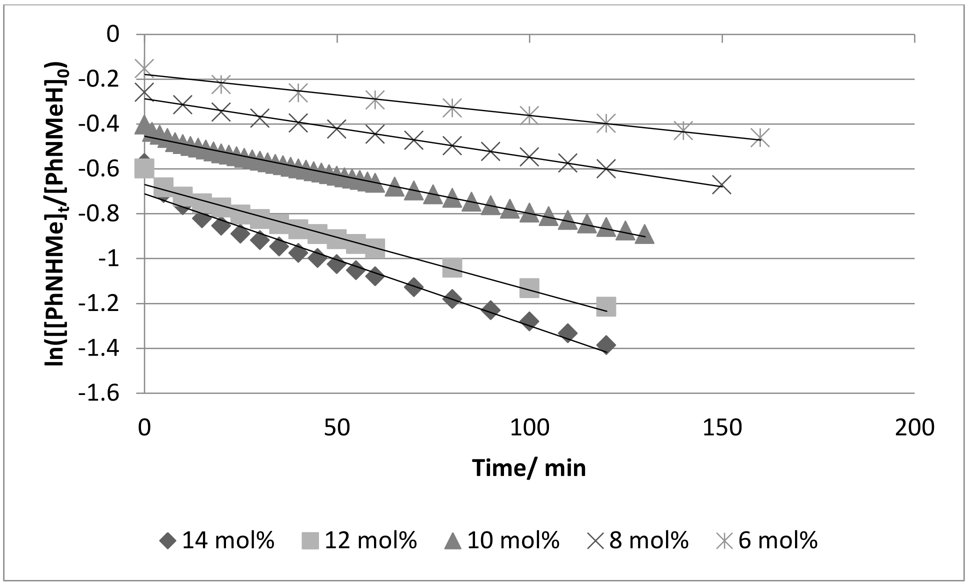

Figure S44: The plot of observed rate constant (k_{obs}) against catalyst loading against [**I**]^2 for a range of **I** concentrations.

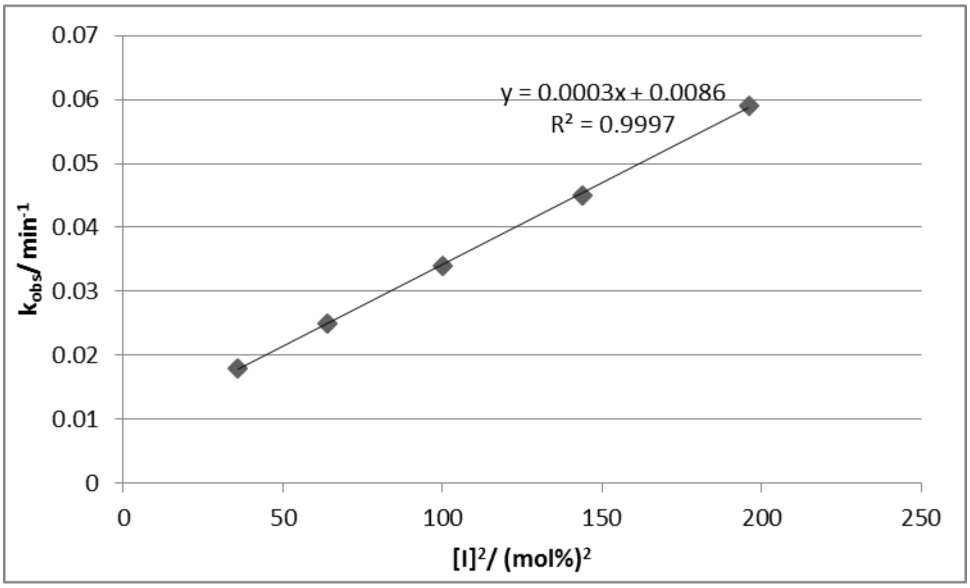

Figure S45: The overall first-order plot of f([amine]$_t$/[amine]$_0$) against time for a reaction with a tenfold excess of pinacol(borane).

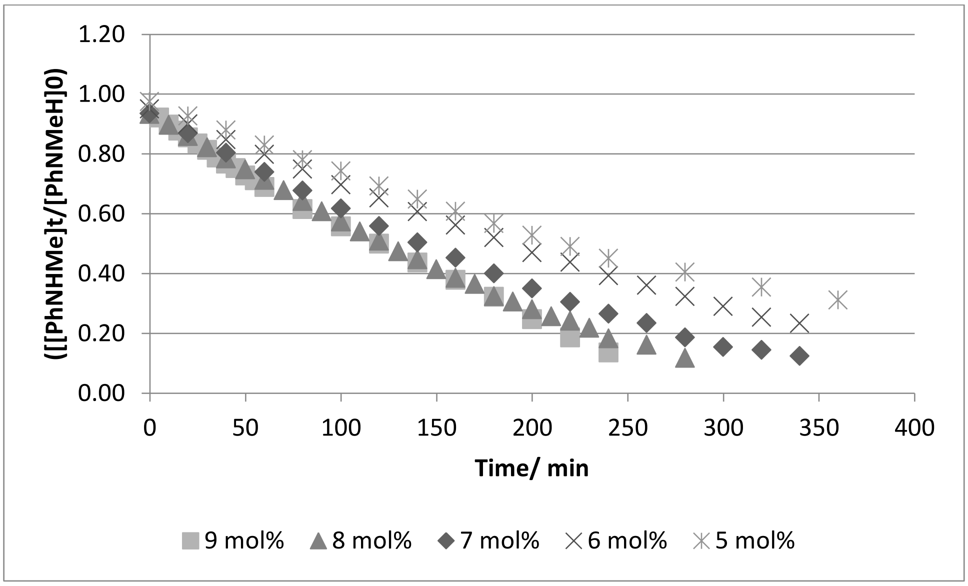

Figure S46: The variable temperature plots for *N*-methylaniline-9-BBN coupling mediated by **I**.

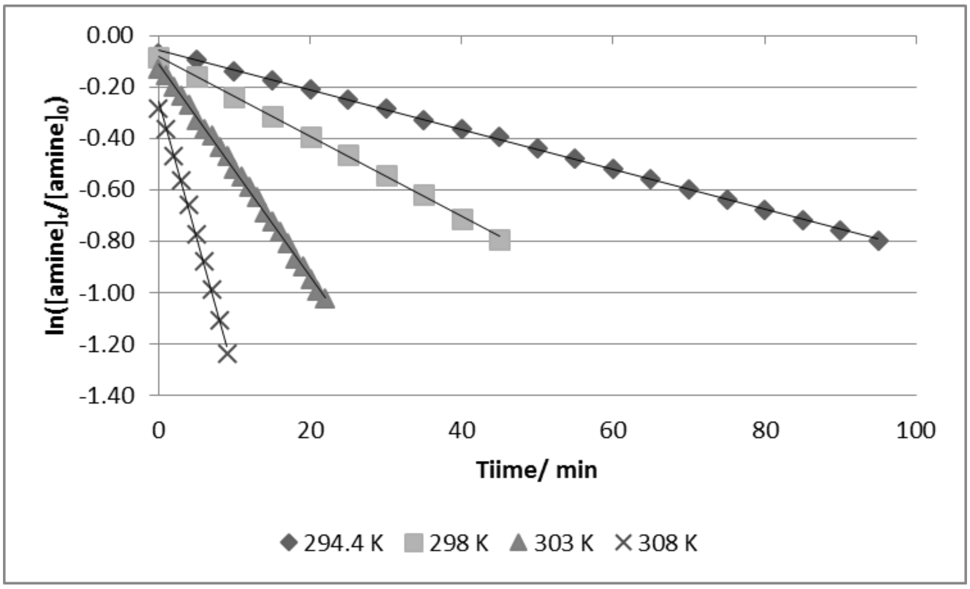

Figure S47: The variable temperature plots for N-methylaniline-pinacol(borane) coupling mediated by **I**.

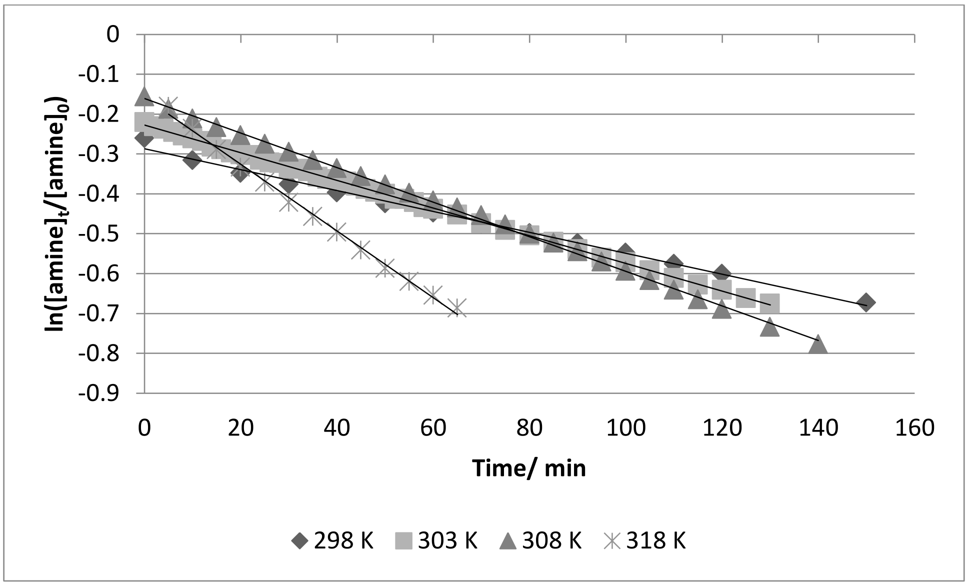

Figure S48: Arrhenius analyses of *N*-methylaniline and borane dehydrocoupling catalyzed by **I**.

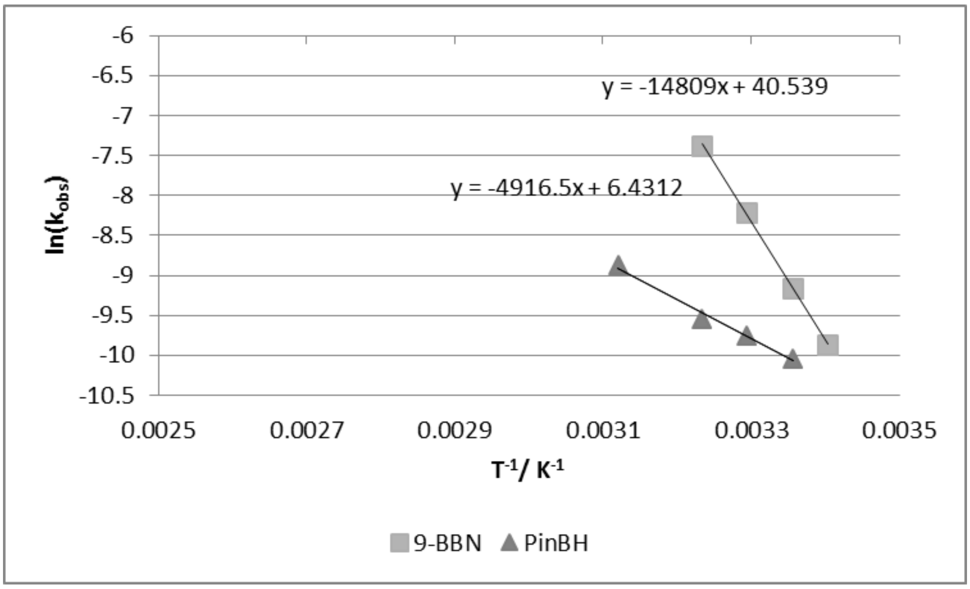

Figure S49: Eyring analyses of *N*-methylaniline borane dehydrocoupling catalyzed by **I**.

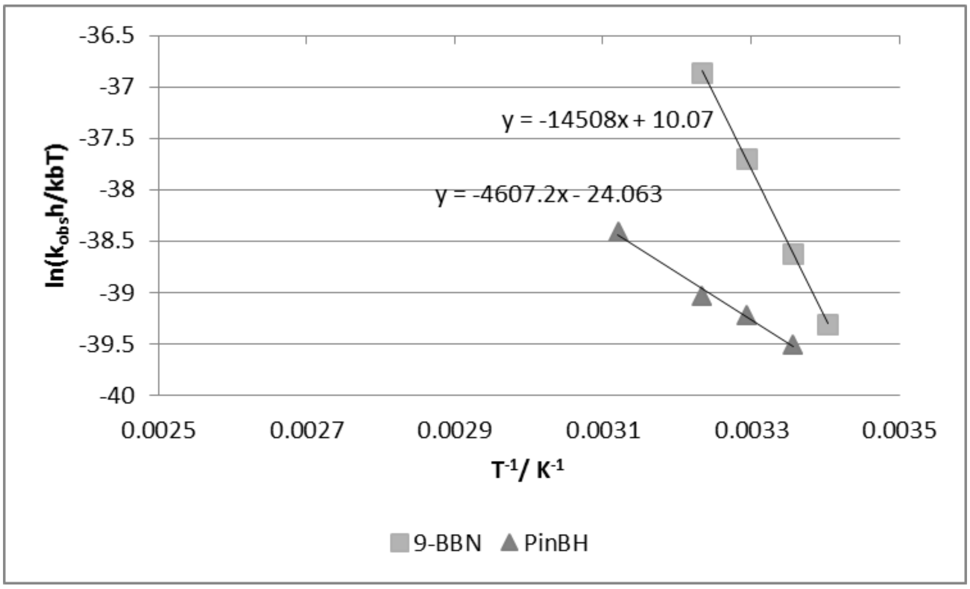

Table S1: Single crystal X-ray diffraction analysis

	**1**	**2**
Empirical formula	$C_{47}H_{70}BMgN_3O_2$	$C_{96}H_{146}B_2Mg_2N_4O_2$
Formula weight (g mol^{-1})	744.18	1457.40
Crystal system	Triclinic	Triclinic
Space group	P-1	P-1
a (Å)	10.4810(4)	10.6157(2)
b (Å)	12.1080(4)	11.7803(3)
c (Å)	18.7980(5)	11.7803(3)
α (°)	94.881(2)	84.5660(10)
β (°)	103.832(2)	84.3360(10)
γ (°)	104.473(2)	84.3360(10)
V (Å)	2216.08(13)	2228.54(8)
Z	2	1
ρ (g cm^{-3})	1.115	1.086
μ (mm^{-1})	0.079	0.075
θ range (°)	5.39 to 25.05	3.07 to 30.07
Measured/independent reflections/ R_{int}	7761 / 5250 / 0.0918	43046 / 12947 / 0.0595
Data / restraints / parameters	7761 / 0 / 505	12947 / 0 / 531
Goodness-of-fit on F^2	1.030	1.019
R_1, wR_2 [I > 2σ(*I*)]	0.0565, 0.1249	0.0514, 0.1182
R_1, wR_2 (all data)	0.0960, 0.1488	0.0837, 0.1356

Data for compounds **1** and **2** were collected on a Nonius Kappa CCD diffractometer equipped with a low temperature device, using graphite monochromated MoKα radiation (λ= 0.71073 Å). Data were processed using the Nonius Software.[4] Structure solution, followed by full-matrix least squares refinement was performed using the programme suite X-SEED throughout.[5] The data for compound **1** were truncated to a Bragg angle of 25° because of a consequent fall-off in diffracting ability that reflects a very small crystal size. The asymmetric unit of compound **2** consists of one molecule of the complex and 2 solvent entities. The THF ligand in the magnesium complex exhibits disorder of C31 and C32 in an 80:20 ratio. Some similarity distance restraints were included for comparative bonds in the disordered regions to assist convergence. The borohydride hydrogen atoms were readily located and refined freely. The solvent moiety based on C61 is present at half occupancy and it straddles a space-group inversion centre. The phenyl ring therein was treated as a rigid hexagon in the refinement. The second solvent moiety is present as a full occupancy, half molecule of toluene, proximate to an inversion centre which serves to generate the remainder. This necessarily means that

the methyl group (C54) is disordered and present at half occupancy. The hydrogen atom with which C54 is disordered was omitted from the refinement.

References

[1] M. Stender, R. J. Wright, B. E. Eichler, J. Prust, M. M. Olmstead, H. W. Roesky, P. P. Power, *J. Chem. Soc., Dalton Trans.* **2001**, 3465.

[2] A. P. Dove, V. C. Gibson, P. Hormnirun, E. L. Marshall, J. A. Segal, A. J. P. White, D. J. Williams, *Dalton. Trans.* **2003**, 3088.

[3] (a) M. H. Chisholm, J. C. Gallucci, K. Phomphrai, *Chem. Commun.*, **2003**, 48; (b) M. H. Chisholm, J. C. Gallucci, K. Phomphrai, *Inorg. Chem.*, **2004**, *43*, 6717.

[4] DENZO-SCALEPACK Otwinowski, Z; Minor, W. "Processing of X-ray Diffraction Data Collected in Oscillation Mode", Methods in Enzymology, Volume 276: Macromolecular Crystallography, part A, p.307-326, **1997**,C.W. Carter, Jr. & R. M. Sweet, Eds., Academic Press.

[5] Barbour, L. J. "X-Seed - A software tool for supramolecular crystallography" *J. Supramol. Chem.* **2001**, *1*, 189-191.
